# Incidence of typhoid fever in Burkina Faso, Democratic Republic of the Congo, Ethiopia, Ghana, Madagascar, and Nigeria (the Severe Typhoid in Africa programme): a population-based study

**DOI:** 10.1016/S2214-109X(24)00007-X

**Published:** 2024-03-12

**Authors:** Florian Marks, Justin Im, Se Eun Park, Gi Deok Pak, Hyon Jin Jeon, Lady Rosny Wandji Nana, Marie-France Phoba, Lisette Mbuyi-Kalonji, Ondari D Mogeni, Biruk Yeshitela, Ursula Panzner, Ligia María Cruz Espinoza, Tigist Beyene, Michael Owusu-Ansah, Sampson Twumasi-Ankrah, Melese Yeshambaw, Ashenafi Alemu, Oluwafemi J Adewusi, Olukemi Adekanmbi, Ellen Higginson, Akinlolu Adepoju, Sarah Agbi, Enoch G Cakpo, Veronica O Ogunleye, Gaëlle Nkoji Tunda, Odion O Ikhimiukor, Jules Mbuyamba, Trevor Toy, Francis Opoku Agyapong, Isaac Osei, John Amuasi, Tsiriniaina Jean Luco Razafindrabe, Tiana Mirana Raminosoa, Gabriel Nyirenda, Njariharinjakampionona Randriamampionona, Hyeong Won Seo, Hyejin Seo, Mohamadou Siribie, Megan E Carey, Michael Owusu, Christian G Meyer, Ndrainaharimira Rakotozandrindrainy, Nimarko Sarpong, Mathilde Razafindrakalia, Ravomialisoa Razafimanantsoa, Moussa Ouedraogo, Yeonseon J Kim, Jooah Lee, Raphaël M Zellweger, Sophie S Y Kang, Ju Yeon Park, John A Crump, Liselotte Hardy, Jan Jacobs, Denise O Garrett, Jason R Andrews, Nimesh Poudyal, Deok Ryun Kim, John D Clemens, Stephen G Baker, Jerome H Kim, Gordon Dougan, Jonathan D Sugimoto, Sandra Van Puyvelde, Aderemi Kehinde, Oluwafemi A Popoola, Vittal Mogasale, Robert F Breiman, William R MacWright, Abraham Aseffa, Birkneh Tilahun Tadesse, Andrea Haselbeck, Yaw Adu-Sarkodie, Mekonnen Teferi, Abdramane Soura Bassiahi, Iruka N Okeke, Octavie Lunguya-Metila, Ellis Owusu-Dabo, Raphaël Rakotozandrindrainy

**Affiliations:** a International Vaccine Institute, Seoul, South Korea; b Faculty of Medicine, The University of Queensland, Brisbane, QLD, Australia; c Cambridge Institute of Therapeutic Immunology and Infectious Disease, University of Cambridge School of Clinical Medicine, Cambridge Biomedical Campus, Cambridge, UK; d Heidelberg Institute of Global Health, University of Heidelberg, Heidelberg, Germany; e Madagascar Institute for Vaccine Research, University of Antananarivo, Antananarivo, Madagascar; f Yonsei University Graduate School of Public Health, Seoul, South Korea; g Institut Supérieur des Sciences de la Population, Ouagadougou, Burkina Faso; h Department of Microbiology, Institut National de Recherche Biomédicale, Kinshasa, Democratic Republic of the Congo; i Department of Medical Biology, Microbiology Service, University Teaching Hospital of Kinshasa, University of Kinshasa, Kinshasa, Democratic Republic of the Congo; j Faculty of Medicine, Congo Protestant University, Kinshasa, Democratic Republic of the Congo; k Armauer Hansen Research Institute, Addis Ababa, Ethiopia; l School of Public Health, Kwame Nkrumah University of Science and Technology, Kumasi, Ghana; m Department of Statistics and Actuarial Science, Kwame Nkrumah University of Science and Technology, Kumasi, Ghana; n Department of Clinical Microbiology, Kwame Nkrumah University of Science and Technology, Kumasi, Ghana; o Department of Medical Diagnostics, Kwame Nkrumah University of Science and Technology, Kumasi, Ghana; p Department of Medicine, University of Ibadan, Ibadan, Nigeria; q Department of Paediatrics, University of Ibadan, Ibadan, Nigeria; r College of Medicine, University of Ibadan, Ibadan, Nigeria; s Department of Community Medicine, University College Hospital, Ibadan, Nigeria; t Department of Pharmaceutical Microbiology, Faculty of Pharmacy, University of Ibadan, Ibadan, Nigeria; u Department of Medical Microbiology and Parasitology, College of Medicine, University of Ibadan, Ibadan, Nigeria; v Department of Community Medicine, College of Medicine, University of Ibadan, Ibadan, Nigeria; w Medical Research Council Unit, The Gambia at London School of Hygiene & Tropical Medicine, Banjul, The Gambia; x Faculty of Infectious & Tropical Diseases, London School of Hygiene & Tropical Medicine, London, UK; y Department of Infection Biology, London School of Hygiene & Tropical Medicine, London, UK; z Bernhard Nocht Institute of Tropical Medicine, Hamburg, Germany; aa International AIDS Vaccine Initiative, Chelsea & Westminster Hospital, London, UK; ab Centre for Health System Strengthening (CfHSS), Kumasi, Ghana; ac Kumasi Centre for Collaborative Research in Tropical Medicine (KCCR), Kumasi, Ghana; ad Institute of Tropical Medicine, Eberhard-Karls University Tübingen, Tübingen, Germany; ae Duy Tan University, Da Nang, Viet Nam; af Hôpital Protestant Schiphra, Ouagadougou, Burkina Faso; ag Centre for International Health, University of Otago, Dunedin, New Zealand; ah Department of Clinical Sciences, Institute of Tropical Medicine, Antwerp, Belgium; ai Department of Microbiology, Immunology and Transplantation, KU Leuven, Leuven Belgium; aj Sabin Vaccine Institute, Washington, DC, USA; ak Division of Infectious Diseases and Geographic Medicine, Stanford University School of Medicine, Stanford, CA, USA; al Jonathan and Karin Fielding School of Public Health, University of California Los Angeles, Los Angeles, CA, USA; am Department of Life Sciences, College of Natural Sciences, Seoul National University, Seoul, South Korea; an Epidemiologic Research and Information Center, Cooperative Studies Program, Office of Research and Development, United States Department of Veterans Affairs, Seattle, WA, USA; ao Department of Epidemiology, University of Washington, Seattle, WA, USA; ap Vaccine and Infectious Disease Division, Fred Hutchinson Cancer Research Center, Seattle WA USA; aq Laboratory of Medical Microbiology, Vaccine & Infectious Disease Institute, University of Antwerp, Antwerpen, Belgium; ar Department of Global Health, Rollins School of Public Health, Emory University, Atlanta, GA, USA; as Infectious Diseases and Oncology Research Institute, University of the Witwatersrand, Johannesburg, South Africa; at Public Health Surveillance Group, Princeton, NJ, USA; au Division of Clinical Pharmacology, Department of Laboratory Medicine, Karolinska Institutet, Karolinska University Hospital Huddinge, Stockholm, Sweden; av Center for Innovative Drug Development and Therapeutic Trials for Africa, College of Health Sciences, Addis Ababa University, Addis Ababa, Ethiopia

## Abstract

**Background:**

Typhoid Fever remains a major cause of morbidity and mortality in low-income settings. The Severe Typhoid in Africa programme was designed to address regional gaps in typhoid burden data and identify populations eligible for interventions using novel typhoid conjugate vaccines.

**Methods:**

A hybrid design, hospital-based prospective surveillance with population-based health-care utilisation surveys, was implemented in six countries in sub-Saharan Africa. Patients presenting with fever (≥37·5°C axillary or ≥38·0°C tympanic) or reporting fever for three consecutive days within the previous 7 days were invited to participate. Typhoid fever was ascertained by culture of blood collected upon enrolment. Disease incidence at the population level was estimated using a Bayesian mixture model.

**Findings:**

27 866 (33·8%) of 82 491 participants who met inclusion criteria were recruited. Blood cultures were performed for 27 544 (98·8%) of enrolled participants. Clinically significant organisms were detected in 2136 (7·7%) of these cultures, and 346 (16·2%) *Salmonella enterica* serovar Typhi were isolated. The overall adjusted incidence per 100 000 person-years of observation was highest in Kavuaya and Nkandu 1, Democratic Republic of the Congo (315, 95% credible interval 254–390). Overall, 46 (16·4%) of 280 tested isolates showed ciprofloxacin non-susceptibility.

**Interpretation:**

High disease incidence (ie, >100 per 100 000 person-years of observation) recorded in four countries, the prevalence of typhoid hospitalisations and complicated disease, and the threat of resistant typhoid strains strengthen the need for rapid dispatch and implementation of effective typhoid conjugate vaccines along with measures designed to improve clean water, sanitation, and hygiene practices.

**Funding:**

The Bill & Melinda Gates Foundation.

## Introduction

Typhoid fever is a febrile illness caused by infection with the Gram-negative bacterium, *Salmonella enterica* serovar Typhi (*S* Typhi). Typhoid fever causes substantial morbidity and mortality in low-income and middle-income countries (LMICs) with limited access to clean water, sanitation facilities, and hygiene.^1^, ^2^ There are between 12·5 million and 16·3 million cases and 140 000 deaths per year due to typhoid.^1^ The most affected regions are sub-Saharan Africa and south Asia.^1^, ^3^, ^4^, ^5^, ^6^, ^7^

The Typhoid Fever Surveillance in Africa Program (TSAP) investigated typhoid fever at 13 sites in ten African countries and reported a high incidence of infection with *Salmonella* spp in both urban and rural settings, with notable variation between sites. Data from TSAP and other similar prospective surveillance studies contributed to the WHO position paper that recommended the introduction of typhoid conjugate vaccines (TCVs) in typhoid-endemic countries.^8^

Typhoid fever presents with non-specific signs and symptoms common to numerous febrile illnesses.^9^ Blood culture is the reference diagnostic method for confirmation of *S* Typhi infection in febrile patients, although sensitivity is only 50–70%.^9^, ^10^ First-line antibiotic treatment of typhoid fever can include ampicillin, chloramphenicol, or cotrimoxazole; however, the emergence of antimicrobial resistance in *S* Typhi isolates has led to increased reliance on other classes of antibiotics, such as fluoroquinolones, third-generation cephalosporins, and azithromycin. If left untreated, infection can progress to severe disease, including intestinal perforation and death.^11^, ^12^ Here we aimed to present estimates of typhoid disease incidence, characterisation of antimicrobial resistance, and evaluation of disease severity from a prospective hospital-based febrile illness surveillance study, the Severe Typhoid in Africa (SETA) programme, conducted in six countries in sub-Saharan Africa.

**Research in context**

**Evidence before this study**

Typhoid fever, resulting from infection by *Salmonella enterica* serovar Typhi, has been largely eliminated in high-income countries with advanced water and sanitation infrastructure. In contrast, typhoid is still a major contributor to morbidity and mortality in low-income and middle-income countries, causing between 12·5 and 16·3 million cases and 140 000 deaths globally per year (Global Burden of Disease, 2017). On May 1, 2017, we searched databases that included PubMed and MEDLINE, MedRxiv, and Embase using the keywords and iterations of “typhoid fever” AND “blood culture” AND “Africa” AND “incidence” in the title and abstract without restriction to language or publication date. Of the 18 results, there was only one prospective study that evaluated population-level blood-culture-confirmed typhoid incidence rates in a multicountry, standardised approach. This was the Typhoid Fever Surveillance in Africa Program, which assessed typhoid in 13 sites in ten sub-Saharan countries. The predominant finding of this study was to describe high adjusted incidences of typhoid in both urban (adjusted incidence of 284 per 100 000 person-years of observation [PYO; 95% credible interval {CI} 217–371] in Kibera, Kenya) and rural settings (adjusted incidence of 373 per 100 000 PYO [274–535] in Polesgo, Burkina Faso), with the highest burden in school-aged children. Multidrug-resistant serotypes were identified at three sites. The degree to which emergent multidrug-resistant infections caused severe health outcomes and the frequency of untreated disease progressing to intestinal perforation were identified as areas for further study. The 2017 Global Burden of Disease reports aggregated studies from the region and estimated the incidence of typhoid fever in Eastern Africa as 726·4 per 100 000 PYO (95% CI 615·6–856·5) and 653·2 per 100 000 PYO (534·8–794·0) in Western Africa. Prospective studies characterising the severity of typhoid fever illness in the region were not identified.

**Added value of this study**

This study introduces new typhoid fever burden estimates from existing and newly established sentinel surveillance centres from six African countries—Burkina Faso, Democratic Republic of the Congo, Ethiopia, Ghana, Madagascar, and Nigeria. Notably, recruitment at tertiary centres extended the scope of clinical presentations to include those with progressive disease and severe complications. The highest incidence of typhoid, estimated per 100 000 PYO, was found in Kavuaya and Nkandu 1, Democratic Republic of the Congo (315, 95% CI 254–390) followed by Imerintsiatosika, Madagascar (162, 126–210). School-aged children, aged 2–15 years, were at high risk across all sites. Through recruitment at centres for which emergency surgery services were available, 157 confirmed hospitalisations for typhoid were recorded. Of these 50 (32%) were classified as severe—defined as confirmed typhoid accompanied by at least one complication. Suspected typhoid intestinal perforations were also high, particularly in the Democratic Republic of the Congo and Ghana, suggesting that a substantial proportion of typhoid infections could be undiagnosed and untreated.

**Implications of all the available evidence**

At the time of this report, five countries, Pakistan, Liberia, Zimbabwe, Nepal, and Malawi have introduced a WHO-prequalified typhoid conjugate vaccine (TCV) into routine immunisation. TCVs have several advantages over live-attenuated (Ty21a) and polysaccharide (ViPS) vaccines, including approval in infants as young as 6 months. Nuances of typhoid illness and evidence of typhoid burden within sub-Saharan Africa have been mounting and clinical studies of TCVs orchestrated by the Typhoid Vaccine Acceleration Consortium and the Typhoid Conjugate Vaccine Introduction in Africa programme have shown promising public health value. Typhoid control programmes using TCV introduction should be prioritised in endemic settings and further research of optimal vaccine delivery strategies should be undertaken.

## Methods

### Study design and population

This study was a prospective, facility-based, hybrid surveillance, population-based study. Eligible participants were screened and recruited from a network of study health centres, which included primary-level, secondary-level, and tertiary-level facilities. To extend our estimation of disease incidence to the general population, health-care utilisation surveys were administered in the catchment population at multiple timepoints to account for differences in health-care-seeking behaviour. Patients who were febrile were recruited from 12 defined study areas in Burkina Faso, Democratic Republic of the Congo, Ethiopia, Ghana, Madagascar, and Nigeria (figure). Sites were selected based on existing evidence of high incidence rates from previous studies (in Burkina Faso, Ghana, Madagascar, the Democratic Republic of the Congo, and Nigeria).

**Figure:**
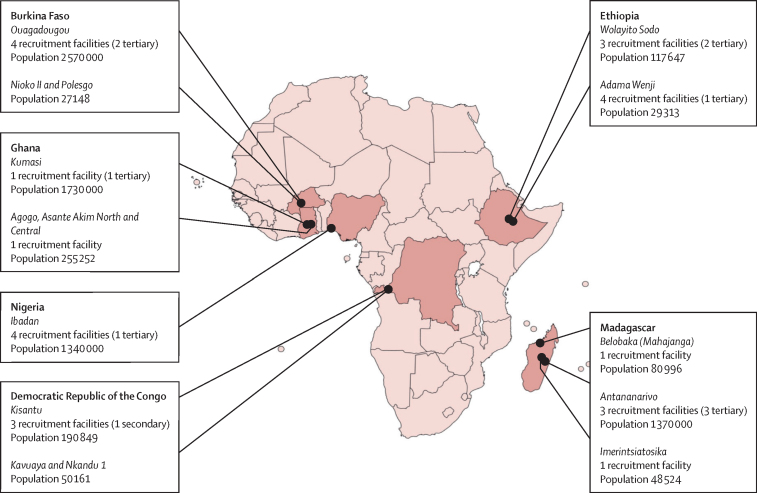
Surveillance sites and study populations in six countries included in the Severe Typhoid in Africa programme

The surveillance catchment area for each site contained two components: a large area that was defined as the administrative area that had access to a study tertiary-level hospital, characterised by the availability of emergency surgical services, and a nested area, which was usually contained within the larger area and was defined as the population that was served by a study primary or secondary-level health centre. In the Democratic Republic of the Congo, only primary and secondary health facilities participated in the surveillance since the Kisantu Hospital Saint Luc centre is a general referral hospital in Kisantu with capacity for emergency surgical services. These populations were not mutually exclusive. Only the population of the nested area was used for estimation of disease incidence. Recruitment at tertiary centres or centres with emergency surgical capacity aimed to identify and characterise severe cases but not contribute to disease incidence calculations. We recruited individuals of all ages who resided in one of the 12 defined catchment areas and visited one of the 25 designated SETA study facilities that included 15 primary or secondary health centres and ten tertiary hospitals (table 1, figure). Notably, recruitment at the Kisantu Hospital Saint Luc centre in the Democratic Republic of the Congo was laboratory-based and patients with a blood culture positive for *Salmonella* spp through routine surveillance were considered for enrolment into the study. Agogo (Ghana), Kisantu (Democratic Republic of the Congo), and Imerintsiatosika (Madagascar) were classified as rural settings. Full study details have been published elsewhere.^7^, ^13^ An overarching scientific advisory group, called the Scientific Advisory Process for Optimal Research on Typhoid provided oversight and recommendations to both Asian (The Surveillance for Enteric Fever in Asia Project) and African (SETA) study sites.^14^

**Table 1:** Countries, study sites, and health-care facilities conducting surveillance for the Severe Typhoid in Africa programme for invasive salmonellosis

	**Health-care facility**	**Health-care facility type**
**Burkina Faso**		
Ouagadougou (urban)	Yalgado Hospital	Tertiary
Ouagadougou (urban)	Charles de Gaulle Hospital	Paediatric tertiary
Ouagadougou (urban)	Kossodo Hospital*	Secondary
Ouagadougou (urban)	Polesgo Health Care Center*	Primary
**Democratic Republic of the Congo**		
Kisantu (rural and urban)	Kisantu Hospital Saint-Luc*	Secondary
Kisantu (rural and urban)	Nkandu 1 Health Center*	Primary
Kisantu (rural and urban)	Kavuaya Health Center*	Primary
**Ethiopia**		
Wolayita Sodo (semi urban)	Sodo Health Center*	Primary
Wolayita Sodo (semi urban)	Sodo Teaching Hospital	Tertiary
Wolayita Sodo (semi urban)	Sodo Christian Hospital	Tertiary
Adama Wenji (semi urban)	Shewa Alem Tena Health Center	Primary
Adama Wenji (semi urban)	Gefersa Health Center	Primary
Adama Wenji (semi urban)	Kuriftu Health Center	Primary
Adama Wenji (semi urban)	Adama Hospital	Tertiary
**Ghana**		
Asante Akim North District and Central Asante Akim Central Municipal (rural and urban)	Agogo Presbyterian Hospital*	Secondary
Kumasi Metropolis (urban)	Komfo Anokye Teaching Hospital	Tertiary
**Madagascar**		
Antananarivo Renivohitra (urban)	Centre Hospitalier Universitaire d'Antananarivo- Hopital Joseph Ravoahangy Andrianavalona	Tertiary
Antananarivo Renivohitra (urban)	Centre Hospitalier Universitaire Joseph Raseta Befelatanana	Tertiary
Antananarivo Renivohitra (urban)	Centre Hospitalier Universitaire Mere Enfant Tsaralalana	Paediatric tertiary
Imerintsiatosika (rural)	Imerintsiatosika Centre de Santé de Base II (CSBII)*	Primary
Belobaka in Mahajanga II (coastal)	Belobaka Centre de Santé de Base II (CSBII)*	Primary
**Nigeria**		
Metropolitan Ibadan (urban)	University College Hospital	Tertiary
Metropolitan Ibadan (urban)	Our Lady of Apostles Catholic Hospital Oluyoro*	Secondary
Metropolitan Ibadan (urban)	Adeoyo Maternity Teaching Hospital*	Secondary
Metropolitan Ibadan (urban)	Kola Daisi Foundation Community Health Centre*	Primary

* Hospitals with access to a discrete catchment area, enabling calculation of adjusted incidences.

In brief, patients who are febrile with either a history of fever (three consecutive days of self-reported fever within the past 7 days) or acute fever (temperature of 37·5°C axillary or 38·0°C tympanic at the time of screening) living in the study area were invited to participate in the study. Following informed consent or assent, a single blood specimen was collected to assess bacterial growth using microbiological cultures. In the Democratic Republic of the Congo, two samples, one from each group, were collected from adult participants. Typhoid fever was defined as a febrile illness in which *S* Typhi was isolated by blood culture. Severe disease was defined as typhoid fever with at least one additional symptom, including gastrointestinal bleeding, gastrointestinal perforation, encephalopathy, meningitis, haemodynamic shock, myocarditis, hepatitis, cholecystitis, pneumonia, pleural effusion, anaemia, focal infection, or renal impairment.^13^ Clinical and demographic data were recorded systematically upon enrolment in the study. Sex was defined as female and male, on the basis of self-report.

The study was approved by the International Vaccine Institute Institutional Review Board (2015-006); the Institute of Tropical Medicine Antwerp Institutional Review Board, Belgium; Universiteit Antwerpen, Comite voor Medische Ethiek, Belgium; Ministère de la Santé du Burkina Faso, Comité d’Ethique pour la Recherche en Santé, Burkina Faso; Comité d’Ethique de l’Ecole de Santé Publique de l’Université de Kinshasa, the Democratic Republic of the Congo (ESP/CE/011/2017); National Research Ethics Review Committee, Ministry of Science and Technology, Ethiopia; Armauer Hansen Research Institute, All African Leprosy, Tuberculosis, and Rehabilitation Training Center Ethics Review Committee, Ethiopia; National Research Ethics Review Committee, Ethiopia; Kwame Nkrumah University of Science and Technology, School of Medical Sciences, Komfo Anokye Teaching Hospital, Committee on Human Research, Publication and Ethics, Ghana; Ministère de la Santé du Repoblikan’l Madagaskar, Comité d’Ethique, Madagascar; University of Ibadan, University College Hospital Ethics Committee, Ibadan, Nigeria (UI/EC/16/0369); Ethics Committee, Our Lady of Apostles Catholic Hospital Oluyoro, Ibadan, Nigeria (OCH/EC/17/05); and Oyo State Ethics Review Committee, Nigeria (AD13/479/665A). Ethical review boards reviewed the study annually to ensure continued compliance with the ethical principles and guidelines from WHO (2009), the Council for International Organizations of Medical Sciences (2016), and the Declaration of Helsinki (World Medical Association Declaration of Helsinki, 2013).

Written informed consent was obtained from all eligible participants or their legal guardians. The informed consent process included an explanation by study staff about the study purpose, expectations from participants, duration of participation, risks and benefits of participation, confidentiality, right to decline or withdraw from the study, and contact information of study investigators. Participants were asked to sign a statement of consent if they agreed to join the study. If the participant was an infant or child, his or her parent or guardian was asked to sign or thumbprint the statement of consent. If the participant was an adolescent, both the participant and the parent or guardian were asked to sign or thumbprint the statement of consent. If the participant was illiterate, an independent literate witness (when possible, this person was to be selected by the participant and have no connection to the study team) was asked to sign or thumbprint the statement of consent.

### Laboratory procedures

Blood (from all recruited patients) and peritoneal fluid (from patients with suspected typhoid intestinal perforation) were cultured using an automated system (BACTEC Peds Plus Medium/BACTEC Plus Aerobic-F, BACTEC, Becton-Dickinson, Franklin Lakes, NJ, USA or BacTAlert PF FAN/BacTAlert FA FAN Aerobic, bioMérieux, Marcy l’Etoile, France) at all sites except for those in the Democratic Republic of the Congo. When SETA started, the Democratic Republic of the Congo had already implemented an effective and sustainable blood culture surveillance system using aerobic incubator and visual inspection. This system had been in place for about 15 years, showing at least equivalent sensitivity to automated culture. The lab decided to continue using this system due to sustainability issues of installing a new system (eg, maintenance costs and disrupting a well established system). At the Democratic Republic of the Congo sites, specimens were inoculated in BacTAlert bottles and incubated at 37°C in an aerobic incubator and were monitored by visual inspection twice a day for up to 7 days. Gram stains were performed from positively flagged bottles and cultured overnight on MacConkey and chocolate agar. The API 20E and 20NE biochemical test kits (bioMérieux, Marcy l’Etoile, France) were used to identify and characterise Enterobacteriaceae and non-Enterobacteriaceae. We considered coagulase-negative *Staphylococcus*, *Corynebacterium* spp, and *Bacillus* spp as contaminants. Further serotyping of *Salmonella* spp was performed using antibody agglutination reactions. Antibiotic susceptibility testing was performed using the Kirby-Bauer disc diffusion method according to Clinical and Laboratory Standards Institute guidelines available at the time of the study.^15^ We considered first-line antibiotics against *S* Typhi infection to be ampicillin, chloramphenicol, and co-trimoxazole. Multidrug-resistant organisms were defined as those that had resistant phenotypes against all first-line antibiotics. At each site, we conducted confirmatory testing of available isolates by quantitative PCR (qPCR) and, if present at the laboratory, whole genome sequencing. Confirmatory qPCR was performed using the STY0201 and *oriC* probe sets as previously described.^16^, ^17^ For whole genome sequencing, genomic DNA was extracted from isolates using the Wizard Plus Genomic Purification kit (Promega, Madison, WI, USA) and sequenced by Eurofins Genomics (Ebersberg, Germany). Raw reads were assembled and the serovar was determined using the Global Health Research Unit pipeline.^18^ These results are published elsewhere.^19^

### Health-care utilisation survey

We conducted a health-care utilisation survey in randomly selected households from each catchment area.^20^ The aim was to determine both actual and potential patterns of health-care-seeking behaviour stratified by age. Detailed methods have been published elsewhere.^20^ To capture any potential seasonal variations in the occurrence of typhoid fever, we conducted two surveys in each setting, one during the rainy season and one during the dry season. In the Democratic Republic of the Congo a single survey was conducted during the dry season due to logistical reasons.

### Statistical analysis

Age-group-stratified incidence rates (younger than 2 years, 2 to 4 years, 5 to 14 years, and 15 years and older) were estimated for each SETA surveillance site. All data and assumptions were synthesised using a mixture model implemented in MultiBUGS.^21^ The model estimated two quantities, with the incidence rate being defined as their ratio multiplied by 100 000. The numerator was the estimated number of health-care-ascertainable, laboratory-confirmable, symptomatic *S* Typhi infections that occurred during the estimated number of person-years of risk contributed by individuals of an age-group living within each SETA surveillance area (denominator).

Available annual census data and population growth projections were used to estimate the size of the catchment population for each SETA health-care facility during each of the years of SETA surveillance. The proportion of the population in each age group was assumed static and equal to what was observed from available census data. Annual growth rate estimates obtained from the World Bank were considered to be measured with uncertainty; an assumption that was incorporated into our mixture model.^22^ For each age group and surveillance site, the person-years of risk was calculated as the sum across the products of the following quantities: the estimated number of individuals of the age group living in the surveillance area and the proportion of the calendar year during which SETA surveillance was operating. Only incidence rates for nested study populations were included in this report because variability in patient referral systems to tertiary centres interfered with systematic enrolment of mild disease.

For each age group and each surveillance site, the observed cumulative number of blood culture-confirmed symptomatic *S* Typhi infections was integrated with information related to the sensitivity of the blood culture for detecting *S* Typhi, including the seasonal probability that a patient who is febrile and seeking care will do so at a SETA-participating facility, the probability of enrolment among the eligible patients who sought care at a SETA facility, and the probability of a blood culture being conducted once enroled. The data inputs and the model assumptions and code are described in detail in the appendix (pp 2–5). In brief, a Bayesian mixture modelling approach integrated data from multiple diverse sources. After a 5000-iteration burn-in phase, 1500 samples were drawn from the posterior distributions of each site-specific and age-group-specific incidence rate by keeping every 50th iteration from each of three 25 000-iteration Markov Chain Monte Carlo chains. The length of the burn-in phase was determined through qualitative assessment of Markov Chain Monte Carlo chain convergence using the Gelman-Rubin statistic, as implemented by MultiBUGS.^21^, ^23^ The adjusted incidence rates represent the mean (95% credible intervals [CIs]) of the sampled iterations.

### Role of the funding source

The funder of the study had no role in study design, data collection, data analysis, data interpretation, or writing of the report.

## Results

25 designated health-care facilities (table 1) in six African countries comprised the surveillance network. The study was conducted between May, 2016, and May, 2020, with a median surveillance period of 34 months (IQR 29–37 months). 82 491 patients met the study inclusion criteria, of whom 27 866 were enrolled and 27 544 (98·8%) received a blood culture; 14 345 (51·5%) of 27 866 participants enrolled were female (table 2). 5629 (20·2%) of subjects enrolled were inpatients.

**Table 2:** Overview of the Severe Typhoid in Africa study recruitment sites and patient enrolment

	Nioko II and Polesgo,*Burkino Faso	**Ouagadougou, Burkino Faso**	**Kisantu,**†**Democratic Republic of the Congo**	Kavuaya and Nkandu 1,*Democratic Republic of the Congo	Wolayito Sodo,*Ethiopia	Adama Wenji,*Ethiopia	Agogo,*Ghana	**Kumasi, Ghana**	**Antananarivo, Madagascar**	Imerinsi-atosika,*Madagascar	Mahajanga,*Madagascar	Ibadan,*Nigeria
Population	27 148	2 570 000	190 849	50 161	117 647	52 770	220 999	1 730 000	1 370 000	48 524	80 996	1 340 000
Surveillance duration, months	43	33	33	29	27	26	35	35	36	49	20	38
Surveillance period (start–end)	May, 2016–January, 2020	September, 2016–June, 2019	September, 2017–May, 2020	January, 2018–May, 2020	July, 2017–September, 2019	August, 2017–September, 2019	May, 2016–April, 2019	May, 2016–April, 2019	July, 2016–June, 2019	February, 2016–February, 2020	June, 2018–January, 2020	February, 2017–May, 2020
Patients meeting inclusion criteria‡	21 750	2304	10 330	13 490	6861	5387	2821	1138	673	4333	484	12 920
Patients not included§	17 278	577	9287	8243	3997	2885	1266	511	226	1628	163	8564
Patients of all ages enrolled	4472	1727	1043	5247	2864	2502	1555	627	447	2705	321	4356
Patients aged <15 years enrolled	2621/4472 (58·6%)	1353/1727 (78·3%)	889/1043 (85·2%)	3765/5247 (71·8%)	977/2864 (34·1%)	766/2502 (30·6%)	913/1555 (58·7%)	619/627 (98·7%)	371/447 (83·0%)	1324/2705 (48·9%)	178/321 (55·5%)	3184/4356 (73·1%)
Median age (IQR), years	10 (3–25)	2 (0–10)	1 (0–5)	6 (2–17)	23 (7–35)	24 (10–36)	10 (4–29)	3 (1–7)	1 (0–7)	15 (5–27)	13 (6–20)	6 (2–16)
Sex, female	2507/4472 (56·1%)	702/1727 (40·6%)	483/1043 (46·3%)	2809/5247 (53·5%)	1592/2864 (55·6%)	1264/2502 (50·5%)	806/1555 (51·8%)	272/627 (43·4%)	202/447 (45·2%)	1386/2705 (51·2%)	171/321 (53·3%)	2151/4356 (49·4%)
Inpatients	57/4472 (1·3%)	1466/1727 (84·9%)	1025/1043 (98·3%)	165/5247 (3·1%)	335/2864 (11·7%)	173/2502 (6·9%)	420/1555 (27·0%)	521/627 (83·1%)	425/447 (95·1%)	14/2705 (0·5%)	1/321 (0·3%)	1027/4356 (23·6%)
Blood cultures performed	4399/4472 (98·4%)	1680/1727 (97·3%)	1039/1043 (99·6%)	5226/5247 (99·6%)	2863/2864 (100%)	2499/2502 (99·9%)	1550/1555 (99·7%)	614/627 (97·9%)	443/447 (99·1%)	2676/2705 (98·9%)	316/321 (98·4%)	4239/4356 (97·3%)
Blood culture positive¶	726/4399 (16·5%)	331/1680 (19·7%)	913/1039 (87·9%)	261/5226 (5·0%)	179/2863 (6·3%)	288/2499 (11·5%)	132/1550 (8·5%)	113/614 (18·4%)	142/443 (32·1%)	163/2676 (6·1%)	36/316 (11·4%)	610/4239 (14·4%)
Positive contaminant growth	510/4399 (11·6%)	265/1680 (15·8%)	7/1039 (0·7%)	25/5226 (0·5%)	76/2863 (2·7%)	140/2499 (5·6%)	21/1550 (1·4%)	68/614 (11·1%)	67/443 (15·1%)	66/2676 (2·5%)	27/316 (8·5%)	267/4239 (6·3%)
Positive clinically significant organism	74/4399 (1·7%)	44/1680 (2·6%)	904/1039 (87·0%)	222/5226 (4·2%)	92/2863 (3·2%)	128/2499 (5·1%)	111/1550 (7·2%)	40/614 (6·5%)	75/443 (16·9%)	96/2676 (3·6%)	8/316 (2·5%)	342/4239 (8·1%)
Positive *S* Typhi	11	2	83	51	7	0	60	15	2	49	1	65
Females positive for *S* Typhi	5/11 (45·5%)	1/2 (50·0%)	37/83 (44·6%)	23/51 (45·1%)	4/7 (57·1%)	0	26/60 (43·3%)	5/15 (33·3%)	1/2 (50·0%)	21/49 (42·9%)	0	27/65 (41·5%)
Inpatients positive for *S* Typhi	1/11 (9·1%)	2/2 (100%)	79/83 (95·2%)	10/51 (19·6%)	1/7 (14·3%)	0	21/60 (35·0%)	15/15 (100%)	2/2 (100%)	0	0	26/65 (40·0%)
Malaria tests performed	4367/4472 (97·7%)	1284/1727 (74·3%)	1038/1043 (99·5%)	5244/5247 (99·9%)	2550/2864 (89·0%)	1725/2502 (68·9%)	1486/1555 (95·6%)	586/627 (93·5%)	439/447 (98·2%)	2705/2705 (100%)	321/321 (100%)	4065/4356 (93·3%)
Positive malaria	1786/4367 (40·9%)	171/1284 (13·3%)	685/1038 (66·0%)	3585/5244 (68·4%)	231/2550 (9·1%)	169/1725 (9·6%)	677/1486 (45·6%)	134/586 (22·9%)	4/439 (0·9%)	40/2705 (1·5%)	51/321 (15·9%)	904/4065 (22·2%)

Data are n or n/N (%) unless otherwise stated. *S* Typhi=*Salmonella enterica* serovar Typhi.

* Nested study population.

† Severe Typhoid in Africa study enrolment was laboratory-based. Patients with a positive *Salmonella* blood culture identified through ongoing routine febrile surveillance were screened and enrolled.

‡ Estimated mean number of eligible patients, using the number of patients enrolled and the estimated probability of enrolment among eligible patients derived from a review of a subset of the paper-based patient registries at each site. The number refused is estimated as the estimated mean number of eligible patients minus the number of enrolled patients.

§ Patients not included due to non-enrolment hours, exceeding daily enrolment numbers, or refusing consent.

¶ The difference between the sum of positive contaminant and positive non-contaminant growth to positive blood culture include missing data and non-identifiable results.

2136 (7·7%) of 27 544 blood cultures were positive for clinically significant organisms (table 2). The highest clinically significant organism positivity rate was observed in Ibadan, Nigeria (342 [8·1%] of 4239), and the lowest rate in Nioko II and Polesgo, Burkina Faso (74 [1·7%] of 4399). Of clinically significant organisms, 346 (16·2%) of 2055 were *S* Typhi; 150 (43·4%) of 346 occurred among female patients. As a proportion of clinically significant organisms, *S* Typhi accounted for 13 (11·0%) of 118 in Burkina Faso, 134 (11·9%) of 1126 in the Democratic Republic of the Congo, seven (3·2%) of 220 in Ethiopia, 75 (49·7%) of 151 in Ghana, 52 (29·1%) of 179 in Madagascar, and 65 (19·0%) of 342 in Nigeria.

The highest overall adjusted incidences were observed in Kavuaya and Nkandu 1, the Democratic Republic of the Congo (315 per 100 000 person-years of observation [PYO], 95% CI 254–390), followed by Imerintsiatosika, Madagascar (162 per 100 000 PYO, 126–210) and Nioko II and Polesgo, Burkina Faso (133 per 100 000 PYO, 110–160; table 3). The lowest overall adjusted incidence was in Sodo, Ethiopia, 16 per 100 000 PYO (95% CI 13–21). By age group, the highest incidence rates were in those aged 2–14 years at all sites. Eight cases overall were observed in infants younger than 2 years (table 3). Rural settings—Imerintsiatosika, Madagascar; Agogo, Ghana; and Kavuaya and Nkandu 1, the Democratic Republic of the Congo—reported the highest typhoid incidence rates.

**Table 3:** Country, site, and age-group-specific incidence rate (95% CI) of blood-culture-confirmed typhoid fever cases per 100 000 person-years of observation*

	**Observed number of cases**†	**Estimated mean number of adjusted**‡**febrile *S* Typhi cases (95% CI)**	**Estimated mean number of person-years of observation (95% CI)**	**Crude incidence of *S* Typhi cases per 100 000 person-years**	**Estimated mean adjusted incidence of febrile *S* Typhi cases per 100 000 person-years (95% CI)**
**Nioko II and Polesgo, Burkina Faso**					
<2 years	0	..§	16 060 (15 520–16 590)	..§	..§
2 to 4 years	2	32 (25–42)	13 780 (13 320–14 230)	14·5	234 (182–309)
5 to 14 years	4	64 (52–80)	31 200 (30 160–32 230)	12·8	206 (166–258)
≥15 years	5	78 (63–98)	69 740 (67 410–72 040)	7·2	111 (90–140)
All	11	174 (145–209)	130 800 (126 400–135 100)	8·4	133 (110–160)
**Kavuaya and Nkandu 1, Democratic Republic of the Congo**					
<2 years	4	38 (29–49)	11 730 (11 350–12 090)	34.1	322 (246–418)
2 to 4 years	6	62 (50–79)	16 390 (15 850–16 890)	36.6	379 (304–476)
5 to 14 years	26	269 (214–338)	49 450 (47 830–50 950)	52.6	544 (432–683)
≥15 years	15	164 (132–208)	91 900 (88 880–94 690)	16·3	179 (143–224)
All	51	533 (433–658)	169 500 (163 900–174 600)	30·1	315 (254–390)
**Sodo, Ethiopia**¶					
<2 years	0	..§	14 840 (14 210–15 410)	..§	..§
2 to 4 years	0	..§	30 380 (29 100–31 570)	..§	..§
5 to 14 years	5	34 (26–44)	93 450 (89 520–97 090)	5·4	36 (28–47)
≥15 years	2	13 (11–17)	151 000 (144 600–156 900)	1·3	9 (7–11)
All	7	47 (37–59)	289 700 (277 500–300 900)	2·4	16 (13–21)
**Agogo, Ghana**					
<2 years	2	22 (16–29)	50 520 (44 670–56 610)	4·0	44 (32–60)
2 to 4 years	8	111 (87–142)	36 410 (32 190–40 790)	22·0	309 (230–403)
5 to 14 years	42	480 (379–617)	155 100 (137 100–173 700)	27·1	314 (238–411)
≥15 years	8	77 (61–100)	370 500 (327 600–415 100)	2·2	21 (16–28)
All	60	690 (547–880)	612 500 (541 500–686 300)	9·8	114 (87–148)
**Imerintsiatosika, Madagascar**					
<2 years	0	..§	14 350 (13 780–14 960)	..§	..§
2 to 4 years	1	4 (3–6)	17 830 (17 120–18 580)	5·6	24 (18–32)
5 to 14 years	16	87 (65–115)	30 500 (29 280–31 780)	52·5	285 (214–380)
≥15 years	32	198 (155–259)	116 300 (111 700–121 200)	27·5	171 (133–225)
All	49	289 (225–377)	179 000 (171 900–186 600)	27·4	162 (126–210)
**Mahajanga, Madagascar**					
<2 years	0	..§	1355 (1296–1417)	..§	..§
2 to 4 years	0	..§	1725 (1650–1804)	..§	..§
5 to 14 years	1	6 (5–8)	5316 (5085–5560)	18·8	110 (84–145)
≥15 years	0	..§	11 740 (11 230–12 280)	..§	..§
All	1	6 (5–8)	20 130 (19 260–21 060)	5·0	29 (22–38)
**Ibadan, Nigeria**					
<2 years	2	293 (60–897)	383 000 (328 900–433 600)	0·5	77 (16–238)
2 to 4 years	10	589 (426–816)	815 400 (700 200–923 100)	1·2	73 (50–106)
5 to 14 years	47	2193 (1816–2687)	2 080 000 (1 786 000–2 355 000)	2·3	106 (83–137)
≥15 years	6	231 (191–279)	5 861 000 (5 033 000–6 636 000)	0·1	4 (3–5)
All	65	3305 (2667–4237)	9 139 000 (7 849 000–10 350 000)	0·7	36 (28–49)

*S* Typhi=*Salmonella enterica* serovar Typhi. CI=credible interval.

* Summarised are 1500 samples from the posterior distributions of each parameter. Samples were drawn from three Markov Chain Monte Carlo chains of 25 000 iterations each, preceded by a 5000-iteration burn in for each chain. Every 50th iteration of the 25 000 was sampled and summarised here. Qualitative assessment of trace plots indicated good mixing for each chain, and the Gelman-Rubin statistic, as implemented by OpenBUGS, indicated convergence of the three chains well before the end of the 5000-iteraction burn in.

† Case counts and incidence rates were not estimated where no *S* Typhi cases were observed by the Severe Typhoid in Africa study surveillance system.

‡ Observed cases were adjusted by health-care seeking behaviour, the probability of recruiting eligible patients, a blood culture diagnosis, and blood culture sensitivity.

§ Indicates that the number of cases and the incidence rate is 0. This is caused by the fact that no blood culture-confirmed typhoid fever cases were captured by the Severe Typhoid in Africa study surveillance in this age-group and at this site.

¶ There were no typhoid cases reported in Adama, Ethiopia.

Of 5629 patients who were admitted to hospital for treatment at or following enrolment, 2469 (43·9%) were female (table 4). All-cause mortality among inpatients was 332 (5·9%), ranging from 33 (3·5%) of 941 in Ghana and 41 (4·0%) of 1027 in Nigeria to 41 (9·3%) of 440 in Madagascar and 104 (8·7%) of 1190 in the Democratic Republic of the Congo. At least one complication occurred in 1319 (23·4%) of 5629 inpatients and 166 (12·6%) of 1319 with complications died during the observation period. Of complications, 381 (28·9%) of 1319 were diagnosed with intestinal perforation (table 4). Out of the 157 hospitalised patients with confirmed typhoid fever, 50 (31·8%) were classified as severe, with all severe cases reported from the Democratic Republic of the Congo (33 [37·1%] of 89), Ghana (eight [22·2%] of 36), and Nigeria (nine [34·6%] of 26; table 4). Five patients with severe confirmed typhoid fever died; four of the patients who died had intestinal perforation and one had encephalopathy. However, this study did not conduct comprehensive verbal autopsies, therefore cause of death related to typhoid was not recorded.

**Table 4:** Severe typhoid fever and frequency of clinical complications among hospitalised patients

		**Burkina Faso (n=1523)**	**Democratic Republic of the Congo (n=1190)**	**Ethiopia (n=508)**	**Ghana (n=941)**	**Madagascar (n=440)**	**Nigeria (n=1027)**	**Total (n=5629)**
Number of hospitalisations in those aged <15 years		1342 (88·1%)	988 (83·0%)	209 (41·1%)	823 (87·5%)	363 (82·5%)	895 (87·1%)	4620 (82·1%)
Sex, female		599 (39·3%)	557 (46·8%)	246 (48·4%)	435 (46·2%)	195 (44·3%)	437 (42·6%)	2469 (43·9%)
Age, years		1 (0–5)	1 (1–7)	22 (3–35)	4 (1–9)	2 (0–7)	5 (1–10)	3 (1–10)
Days of hospitalisation		4 (2–8)	11 (8–16)	5 (3–9)	4 (2–8)	5 (2–9)	4 (2–6)	6 (3–10)
Overall all-cause mortality		87 (5·7%)	104 (8·7%)	26 (5·1%)	33 (3·5%)	41 (9·3%)	41 (4·0%)	332 (5·9%)
Hospitalisation with at least one complication		168 (11·0%)	311 (26·1%)	158 (31·1%)	238 (25·3%)	166 (37·7%)	278 (27·1%)	1319 (23·4%)
Intestinal perforation		44/168 (26·2%)	206/311 (66·2%)	10/158 (6·3%)	37/238 (15·5%)	2/166 (1·2%)	82/278 (29·5%)	381/1319 (28·9%)
Death in patients reporting complications		26/168 (15·5%)	59/311 (19·0%)	14/158 (8·7%)	19/238 (8·0%)	20/166 (12·0%)	28/278 (10·1%)	166/1319 (12·6%)
Number of hospitalisations with culture-confirmed typhoid fever		3*	89*	1†	36*	2*	26†	157†
Overall deaths		1/3 (33·3%)	5/89 (5·6%)	0	1/36 (2·8%)	0	0	7/157 (4·5%)
Severe typhoid fever cases‡		0	33/89 (37·1%)	0	8/36 (22·2%)	0	9/26 (34·6%)	50/157 (31·8%)
Sex, female		..	12/33 (36·4%)	..	2/8 (25·0%)	..	3/9 (33·3%)	17/50 (34·0%)
Frequency of complications§								
	Gastrointestinal bleeding	..	2/33 (6·1%)	..	1/8 (12·5%)	..	0	3/50 (6·0%)
	Intestinal perforation	..	23/33 (69·7%)	..	4/8 (50·0%)	..	3/9 (33·3%)	30/50 (60·0%)
	Encephalopathy	..	2/33 (6·1%)	..	0	..	0	2/50 (4·0%)
	Meningitis	..	0	..	0	..	2/9 (22·2%)	2/50 (4·0%)
	Hepatitis	..	1/33 (3·0%)	..	0	..	0	1/50 (2·0%)
	Cholecystitis	..	6/33 (18·2%)	..	0	..	1/9 (11·1%)	7/50 (14·0%)
	Pneumonia	..	2/33 (6·1%)	..	2/8 (25·0%)	..	2/9 (22·2%)	6/50 (12·0%)
	Anaemia	..	2/33 (6·1%)	..	2/8 (25·0%)	..	5/9 (55·6%)	9/50 (18·0%)
	Renal impairment	..	0	..	1/8 (12·5%)	..	1/9 (11·1%)	2/50 (4·0%)
	Others	..	6/33 (18·2%)	..	1/8 (12·5%)	..	0	7/50 (14·0%)
Death among severe typhoid fever cases		..	5/33 (15·2%)	..	0	..	0	5/50 (10·0%)

Data are n (%), n/N (%), or median (IQR).

* Confirmed by blood culture only.

† Confirmed by blood, stool, peritoneal fluid, or tissue culture.

‡ Defined as a confirmed typhoid case accompanied by the presence of at least one of gastrointestinal bleeding, gastrointestinal perforation, encephalopathy, meningitis, haemodynamic shock, myocarditis, hepatitis, cholecystitis, pneumonia, pleural effusion, anaemia, focal infection, or renal impairment.

§ Haemodynamic shock, myocarditis, pleural effusion, and focal infection were not reported in any country.

Of the 346 *S* Typhi isolates, more than half of those tested were resistant to ampicillin (172 [57%] of 302). 46 (16%) of 280 tested isolates showed ciprofloxacin non-susceptibility, and sites with the highest occurrence of typhoid, the Democratic Republic of the Congo and Ghana, reported the highest prevalence of ciprofloxacin non-susceptibility: 23 (24%) of 94 tested in the Democratic Republic of the Congo and 13 (18%) of 71 tested in Ghana (table 5). Settings with the highest proportion of ampicillin resistance were Nigeria (48 [94%] of 51) and the Democratic Republic of the Congo (95 [79%] of 120). Co-trimoxazole resistance was highest in Nigeria (37 [77%] of 48), the Democratic Republic of the Congo (76 [72%] of 106), and Burkina Faso (seven [70%] of 10; table 5). Of 264 isolates tested against ampicillin, co-trimoxazole, and chloramphenicol, 43 (16%) showed resistance to all three antibiotics (multidrug resistant); 18 (42%) of 43 of multidrug-resistant isolates were identified among female patients. These isolates were identified in the Democratic Republic of the Congo, Ghana, and Nigeria.

**Table 5:** Antimicrobial resistance profile of *S* Typhi isolates

		**Burkina Faso**	**Democratic Republic of the Congo**	**Ethiopia**	**Ghana**	**Madagascar**	**Nigeria**	**All**
Number of *S* Typhi isolates		13	134	7	75	52	65	346
Resistant organisms*								
	Ampicillin†	0	95/120 (79%)	3/4 (75%)	18/71 (25%)	8/52 (15%)	48/51 (94%)	172/302 (57%)
	Co-trimoxazole†	7/10 (70%)	76/106 (72%)	..	21/67 (31%)	8/52 (15%)	37/48 (77%)	149/283 (53%)
	Chloramphenicol†	5/11 (45%)	26/114 (23%)	0	20/71 (28%)	0	6/53 (11%)	57/306 (19%)
	Ciprofloxacin‡	0	23/94 (24%)	0	13/71 (18%)	4/52 (8%)	6§/53 (11%)	46/280 (16%)
	Ceftriaxone or cefotaxime	0	9/111 (8%)	0	2/70 (3%)	0	3/51 (6%)	14/296 (5%)
	Amoxicillin-clavulanic acid	0	2/2 (100%)	1/2 (50%)	12/68 (18%)	9/52 (17%)	7/44 (16%)	31/180 (17%)
	Tetracycline	..	..	4/5 (80%)	13/69 (19%)	8/51 (16%)	36/45 (80%)	61/170 (36%)
Multidrug resistance¶		0	23/102 (23%)	..	15/67 (22%)	0	5/40 (13%)	43/264 (16%)
	Sex, female	0	12/23 (52%)	0	5/15 (33%)	0	1/5 (20%)	18/43 (42%)
Multidrug resistance‡ and ciprofloxacin (or nalidixic acid) resistance		0	6/85 (7%)	..	3/66 (5%)	0	0	9/241 (4%)
Multidrug resistance‡ and ceftriaxone or cefotaxime		0	3/94 (3%)	..	0	0	0	3/249 (1%)

*S* Typhi=*Salmonella enterica* serovar Typhi.

* As determined by the Clinical and Laboratory Standards Institute breakpoints for which both intermediate and resistant classifications are considered as resistant.

† Conventional first-line antibiotics against *S* Typhi infection.

‡ Isolates with intermediate or resistant characteristics were classified as ciprofloxacin non-susceptible; where ciprofloxacin testing was unavailable, nalidixic acid results were used (see Nigeria).

§ Of six samples with ciprofloxacin non-susceptible phenotypes noted in the table, four were classified based on resistance to nalidixic acid.

¶ Resistant to ampicillin, cotrimoxazole, and chloramphenicol.

## Discussion

Our findings indicate that typhoid fever remains a major cause of differentiated febrile illness in sub-Saharan Africa. Many of the observed incidence rates were higher than the 100 cases per 100 000 PYO, a high burden of disease. Consistent with TSAP findings, we identified the highest burden of *S* Typhi in rural settings, which contrasts with our current understanding of typhoid fever in Asia where typhoid burden is highest in impoverished populations of densely populated urban areas.^5^, ^6^ SETA typhoid incidence estimates by age group were also similar to findings from TSAP. The highest typhoid incidence was reported in children younger than 15 years across sites, consistent with previous estimates from Burkina Faso, Ghana, and rural Madagascar.^6^

Our observation of high typhoid burden in rural settings underscores the need to expand surveillance beyond urban centres despite resource and logistical limitations. The use of serosurveys could provide insight into the extent of typhoid burden in rural areas and inform strategic introduction of TCVs in endemic areas.^24^, ^25^ Typhoid hospitalisation rates varied across sites. These variations might be related to the virulence factors of locally circulating strains but also to factors affecting health service accessibility and quality in each population.

The incidence of typhoid in Ethiopia—a country that has undergone rapid urbanisation and major population migration—was below the threshold for high typhoid burden in both TSAP and this study. Although this study did not identify typhoid fever as a significant cause of febrile illness at study sites in Ethiopia, this does not rule out the possibility that endemic typhoid exists in other regions within the country.^26^, ^27^, ^28^ Whether the low incidence and prevalence of *S* Typhi infection in Ethiopia represents the actual burden of the disease or the poor sensitivity of the blood cultures in this setting is unclear. Ethiopia provides mass administration of azithromycin in areas with high levels of endemic trachoma; exposure to azithromycin can interfere with blood culture sensitivity.^29^, ^30^ These findings will need to be further examined using new environmental surveillance tools and more specific serosurveys.^24^, ^31^, ^32^ Of particular note, serosurveys designed to identify asymptomatic infections and individuals with false-negative blood culture results could provide a more realistic estimate of the burden of typhoid fever in these settings.^31^

Resistance of *S* Typhi isolates to first-line antibiotics was common in most settings, and multidrug-resistant *Salmonella* isolates were common in the Democratic Republic of the Congo, Nigeria, and Ghana. Given the overuse of uncontrolled and widely available over-the-counter antibiotics, antimicrobial resistance will continue to pose a major challenge to those engaged in treating a wide variety of infectious diseases. TCVs have shown good efficacy against typhoid fever and could be an important tool to curb transmission of *S* Typhi bacteria and ease the consumption of antibiotics.^33^ TCVs might also be effective at eliminating the asymptomatic carrier state and, thus, could limit the rate of disease transmission.

The findings of this study should be interpreted while considering several important limitations. First, our estimates of typhoid burden use multiple adjustment coefficients that are designed to compensate for health-seeking behaviour and recruitment efficiency of study health facilities. These variables themselves are subject to uncertainty and are susceptible to biases and errors related to the method and accuracy of data collection. As such, we have observed in some estimates, such as those reported for Burkina Faso, that a low number of positive cases can translate to a high adjusted incidence estimate, although wide credible intervals signal the degree of uncertainty and do not exclude the possibility of moderate incidence in this setting. Second, adjusted incidence stratified by sex could not be estimated due to an absence of data stratified by the variable sex in the denominator. Third, a sensitivity analysis describing the varying effects of our model assumptions was not performed. Such an analysis would inform the degree to which variance in model input variables modified incidence rate estimates; however, inclusion of additional analyses in this report would diminish the primary goal of the report, which was to provide a preliminary description of the burden of typhoid in parts of Africa. Fourth, although prospective surveillance of defined cohorts is the recommended method for estimating typhoid burden, the limited size and idiosyncrasies of each catchment population limit the extent to which findings can be extrapolated to other settings. Assessing the need for TCV introduction within a country might require layered evidence, such as evaluation of typhoid seroprevalence. Fifth, the testing and reporting of antimicrobial susceptibility in all sites was not uniform, limiting the ability of comparative analysis of *S* Typhi isolates between sites. Additionally, the observation of several unexpected results, such as the high proportion of ceftazidime resistance in Madagascar (30 [61%] of 49, not reported) despite low ampicillin resistance (eight [15%] of 52), underscores the need for further confirmatory testing, such as genomic analysis.

This study found that typhoid fever is a major cause of febrile illness in Africa. Increased rates of antimicrobial resistance could eventually render typhoid fever untreatable; this has already been reported for the extensively drug-resistant isolates isolated in Pakistan.^34^ There is a crucial need to introduce TCVs in areas for which there is a high burden of disease; these vaccines will likely contribute substantially to efforts to control typhoid fever. Liberia, Zimbabwe, Nepal, Pakistan, and Malawi have already introduced TCVs into their routine immunisation programmes.^35^, ^36^, ^37^, ^38^ Other African countries, including Ghana, Madagascar, and the Democratic Republic of the Congo are using TCVs as part of ongoing projects.^39^, ^40^ National Immunization Technical Advisory Groups from other African countries, ie, Burkina Faso, Kenya, and Zambia have recommended TCV introduction and further introduction planning efforts are underway.^41^ Two TCVs are already WHO-prequalified and available through Gavi, the Vaccine Alliance's funding mechanism and a third vaccine is undergoing a WHO-prequalification review.^42^, ^43^ Future studies should focus on generating specific data requested by high-risk countries that would allow decision makers to evaluate the need for and the strategic roll out of effective typhoid vaccines.

## Data sharing

The study protocol has been published elsewhere^13^ and is available to download for free. Sharing of the study data will be considered after publication of the Article by request to the corresponding author and with a signed data access agreement.

## Declaration of interests

We declare no competing interests.
