# Correction to *Lancet Glob Health* 2024; 12: e599–610

**DOI:** 10.1016/S2214-109X(24)00136-0

**Published:** 2024-04-09

*Marks F, Im J, Park SE, et al. Incidence of typhoid fever in Burkina Faso, Democratic Republic of the Congo, Ethiopia, Ghana, Madagascar, and Nigeria (the Severe Typhoid in Africa programme): a population-based study.* Lancet Glob Health *2024;* 12: *e599–610*—In this Article, the affiliation for Ashenafi Alemu should have been Armauer Hansen Research Institute, Addis Ababa, Ethiopia. This change has been made as of April 9, 2024.

