# Supplementary material for: Incidence of typhoid fever in Burkina Faso, Democratic Republic of the Congo, Ethiopia, Ghana, Madagascar, and Nigeria (the Severe Typhoid in Africa programme): a population-based study
Source: Lancet Glob Health. 2024 Mar 12;12(4):e599–610. doi: 10.1016/S2214-109X(24)00007-X (PMC10951957; doi:10.1016/S2214-109X(24)00007-X)
Supplement: Supplementary appendix [file mmc1.pdf]

THE LANCET
Global Health

Supplementary appendix

This appendix formed part of the original submission and has been peer reviewed. We post it as supplied by the authors.

Supplement to: Marks F, Im J, Park SE, et al. Incidence of typhoid fever in Burkina Faso, Democratic Republic of the Congo, Ethiopia, Ghana, Madagascar, and Nigeria (the Severe Typhoid in Africa programme): a population-based study. *Lancet Glob Health* 2024; **12:** e599–610.

Supplementary material.

Table of contents

A. Description of the statistical model for estimating incidence rates……………………………………………….2
B. Supplementary Table(s)………………………………………………………………………………………...4
C. MultiBUGS code
C1. Code for model……………………………………………………………………………………………...5
C2. Code for each site's dataset…………………………………………………………………………………9
C3. Batch script for conducting the whole analysis in MultiBUGS……………………………………………14
C4. R code for post-processing the MultiBUGS analysis output………………………………………………18

A. Description of the statistical modeling approach for estimating the incidence of healthcare-ascertainable, laboratory-confirmable, symptomatic *S.* Typhi, by age-group and SETA surveillance site

Age group-stratified incidences rates were estimated for each SETA surveillance site. Data from different sources were synthesized using a mixture modeling approach, which combined population census estimates/projections obtained from the United Nations Development Program (UNDP) with information from study-conducted healthcare utilization surveys and healthcare-facility based disease surveillance activities. Only incidence rates for nested study populations were included in this report because variability in patient referral systems to tertiary centers interfered with systematic enrollment of milder disease. The incidence of healthcare-ascertained, laboratory-confirmable, symptomatic *S.* Typhi was estimated for the nested surveillance areas by country and age groups (under 2 years, 2 to <5 years, 5 to <15 years, and 15 years and older).

Data elements. The available data inputs included an estimate of the size of the catchment population for surveillance site *s*, $P^{h(s)}$, from one calendar year *h* during or close in time to the site's SETA surveillance period (*ymin(s) to ymax(s)*). Estimates of l_y^{y+1}, the year-to-year population growth rate (%) for the contiguous set of calendar years encompassing *h* and the SETA surveillance period for each site, were obtained from the World Bank's population.[1] For each age group *a* at site *s* during season *r* (0=dry; 1=rainy), over the course of SETA surveillance $X_{s,a}^r$ participants were enrolled, $M_{s,a}^r$ of whom had an available blood-culture test result and $F_{s,a}^r$ were blood-culture confirmed symptomatic *S.* Typhi cases. To estimate the proportion of the eligible patients seen at a SETA participating facility who were enrolled, a systematic review was conducted of the paper-based patient admission logbooks maintained by each SETA healthcare facility. This systematic review covered only a subset of the years during which SETA surveillance at each site *s*. From this review, N_{elig}^s and N_{enroll}^s representing the number of SETA eligible and enrolled patients, respectively, were identified. Note, that N_{enroll}^s will not necessarily equal the sum of all $X_{s,a}^r$ for a site. Among the respondents to the healthcare utilization survey who indicated that they would seek care for any fever ($H_{s,a}$), $G_{s,a}$ denotes the number who would have done so at a SETA facility. At the country (c) level (for example, for the three SETA surveillance sites in Burkina Faso), the number of respondents who would seek care for any fever, $K_{c,a}^r$, and who would have done so at a SETA facility, $J_{c,a}^r$, were derived from this healthcare utilization survey.

Mixture model. The age-group specific incidence rate of healthcare-ascertainable, laboratory-confirmable, symptomatic *S.* Typhi, $\hat{I}_{s,a}$, for each site, *s*, where SETA surveillance was conducted during year, *y*, was estimated by $\hat{I}_{s,a} = \frac{\hat{Y}_{s,a}}{\sum_{y=ymin(s)}^{ymax(s)} \hat{P}_{s,a,y}} * 100{,}000$. $\sum_{y=ymin(s)}^{ymax(s)} \hat{P}_{s,a,y}$ and $\hat{Y}_{s,a}$ represent the estimated cumulative number of person-years and healthcare-ascertainable, laboratory-confirmable, symptomatic *S.* Typhi cases, respectively, for a particular combination of surveillance site and age-group.

Estimating $\hat{P}_{s,a,y}$. Here, $\hat{P}_{s,a,y} = P^{h(s)} * g^{s,y} * u_{s,a}^h * p_y^s$. The population growth function is defined as follows for each site $g^{s,y} = \begin{cases} y = h & 0 \\ y < h & \prod_{t=y}^{h}(1 - l_t^{t+1}) \\ y > h & \prod_{t=y}^{h}(1 + l_t^{t+1}) \end{cases}$. As shown above, the proportion of the site's catchment population in age group *a* is assumed fixed for the period of time that SETA surveillance was active and to be equal to that observed at the most recent census. p_y^s is defined as the proportion of calendar year *y* that SETA surveillance was conducted for surveillance site *s*. l_y^{y+1} are each assumed to be drawn from a normal distribution with mean l_y^{y+1} and 1/variance following a gamma distribution with shape and rate parameters equal to 1.0%.

Estimating $\hat{Y}_{s,a}$. The probability $\gamma_{s,a}^r$ that a member of the SETA catchment population of site *s* in age group *a* would seek care at a SETA healthcare facility, as opposed to a non-SETA facility, for a laboratory-confirmable symptomatic *S.* Typhi during season *r* is assumed to follow a logistic distribution with mean and variance equal to

$$log_e\left(\frac{G_{s,a}/H_{s,a}}{1-\frac{G_{s,a}}{H_{s,a}}}\right) + log_e\left(\frac{J_{c,a}^r/(J_{c,a}^{r=0}+J_{c,a}^{r=1})}{(K_{c,a}^r-J_{c,a}^r)/\left((K_{c,a}^{r=0}+K_{c,a}^{r=1})-(J_{c,a}^{r=0}+J_{c,a}^{r=1})\right)}\right)$$ and

$\frac{\left({G_{s,a}}/{H_{s,a}}\right)^2}{3} + \left[\frac{1}{J^r_{c,a}} + \frac{1}{(J^{r=0}_{c,a}+J^{r=1}_{c,a})} + \frac{1}{(K^r_{c,a}-J^r_{c,a})} + \frac{1}{\left((K^{r=0}_{c,a}+K^{r=1}_{c,a})-(J^{r=0}_{c,a}+J^{r=1}_{c,a})\right)}\right]$, respectively. The probability d_s that an eligible patient seeking care at a SETA facility will be enrolled is assumed to follow a logistic distribution with mean $log_e\left(\frac{N^S_{enroll}/N^S_{elig}}{1-\frac{N^S_{enroll}}{N^S_{elig}}}\right)$ and variance $\frac{\left(N^S_{enroll}/N^S_{elig}\right)^2}{3}$. The probability $\alpha^r_{s,a}$ that an enrolled SETA participant with a blood culture ordered at the time of enrollment is positive for *S.* Typhi is assumed to follow a logistic distribution with mean $\frac{F^r_{s,a}}{M^r_{s,a}}$ and variance $\frac{\left(\frac{F^r_{s,a}}{M^r_{s,a}}\right)^2}{3}$. Blood culture is assumed to have an imperfect sensitivity π for detecting laboratory-confirmable symptomatic *S.* Typhi, which is assumed to follow a normal distribution with mean of 60% and standard deviation equal to 5%.

The total number of laboratory-confirmable symptomatic *S.* Typhi cases, $\hat{Y}_{s,a}$, is estimated by the following summation of $\sum_{r=0}^{1}\sum_{b=0}^{1}\sum_{w=0}^{1}\sum_{j=0}^{1}\hat{Z}^{r,b,w,j}_{s,a}$, where $\hat{Z}^{r,b,w,j}_{s,a}$ denotes the estimated number of laboratory-confirmable symptomatic *S.* Typhi cases for each combination of season *r*, blood-culture status *b* (1, cultured; 0, not cultured), whether (1) or not (0) care would be sought for this disease episode at a SETA (*w*), and whether (1) or not (0) those seeking care at a SETA facility were enrolled (*j*). For otherwise-eligible patients who would be expected to seek care at a non-SETA healthcare facility, the value for *j* would be 0. The expectation for $\hat{Z}^{r,b,j,w}_{s,a}$ is as follows,

$$\hat{Z}^{r,b,w,j}_{s,a} = \begin{cases} F^r_{s,a}/\pi & b = 1, j = 1, w = 1 \\ \left(X^r_{s,a} - M^r_{s,a}\right) * \frac{\alpha^r_{s,a}}{\pi} & b = 0, w = 1, j = 1 \\ \left(\frac{X^r_{s,a}}{d_s} - X^r_{s,a}\right) * \frac{\alpha^r_{s,a}}{\pi} & b = \{0,1\}, w = 1, j = 0 \\ \frac{X^r_{s,a}*(1-\gamma^r_{s,a})}{d_s*\gamma^r_{s,a}} * \frac{\alpha^r_{s,a}}{\pi} & b = \{0,1\}, w = 0, j = 0 \end{cases}.$$

A Bayesian mixture modeling approach was employed to draw samples from the posterior distributions of $\hat{Y}_{s,a}$. MultiBUGS (MRC Biostatistics Unit, University of Cambridge, version 2.0) was used to draw 1,500 samples from the posterior distributions of interest.[1] Samples were drawn from three MCMC chains of 25,000 iterations each, following a 5,000-iteration burn-in phase for each chain. Every 50th iteration of each 25,000-iteration chain was retained to summarize posterior distributions. Qualitative assessment of inter-chain mixing was assessed by the Gelman-Rubin statistic as implemented by MultiBUGS, was used to determine the length of the burn-in phase.[3] The adjusted rates represent the mean of the sampled iterations followed by 95% credible intervals. Preparation and post-processing of MultiBUGS analysis results were conducted in R (version 2.1.12 (2021-11-01), R Foundation for Statistical Computing, Vienna, Austria). The 'R2MultiBUGS' R library was not used for this work, but this package could have been used to further streamline this analysis' workflow.

References

1. The World Bank Data: Population Growth (annual %). https://data.worldbank.org/indicator/SP.POP.GROW (Accessed. November 2019)

2. Goudie, R. J. B., Turner, R. M., De Angelis, D., Thomas, A. MultiBUGS: A parallel implementation of the BUGS modelling framework for faster Bayesian inference. *Journal of Statistical Software* 2020; **95** (7). doi:10.18637/jss.v095.i07

3. Brooks S.P., Gelman A. Alternative methods for monitoring convergence of iterative simulations. *Journal of Computational and Graphical Statistics* 1998; **7**:434-455.

B. Supplementary Tables

Table S.B1. Description of the data inputs for the SETA *S.* Typhi incidence rate model.

		Burkina Faso	Democratic Republic of the Congo†	Ethiopia	Ghana	Madagascar		Nigeria
		Nioko and Polesgo	Kavuaya and Nkandu 1	Sodo	Agogo	Imerintsiatosika	Mahajanga	Ibadan
**Parameter**	**Data source**							
$P^{h(s)}$, h	UNDP	27,148; 2017	54,731; 2017	120,288; 2017	140,694; 2010	44,669; 2016	5,475; 2016	1,343,147; 2006
$u^h_{s,a}$	Census	(0.12,0.11,0.24,0.53)	(0.07,0.10,0.29,0.54)	(0.05,0.10,0.32,0.52),	(0.08,0.06,0.25,0.60)	(0.08,0.10,0.17,0.65)	(0.07,0.09,0.26,0.58)	(0.04,0.09,0.23,0.64)
l_y^{y+1}, (%); Start year; End year;	World Bank [World Bank 2019]	(2.9,0,2.9,2.9*,2.9*); 2016; 2020	(3.3,3.2,3.2); 2017; 2019;	(2.7,2.6,2.6); 2017; 2019;	(1,2.5,2.4,2.4,2.3,2.3,2.2,2.2,2.1,2.1); 2010; 2019;	(1,1.6,1.6,1.6); 2016; 2019;	(1,1.6,1.6,1.6); 2016; 2019;	(1.0,4.8,4.8,4.8,4.7,4.7,4.6,4.6,4.5,4.4,4.4,4.3,4.23,4.23,4.23); 2016; 2020;
p_y^s	SETA	(0.66,1,1,1,1)	(1,1,1)	(0.33,1,1)	(0,0,0,0,0,0,0.66,1,1,1)	(0.91,1,1,1)	(0.58,1,1,1)	(0,0,0,0,0,0,0,0,0,0,0.91,1,1,1)
$X^r_{s,a}$ ‡	SETA	Dry: (454, 516, 548, 947) Rainy: (302, 352, 449, 904)	Dry: (469, 425, 578, 671) Rainy: (681, 682, 930, 811)	Dry: (126, 164, 190, 916) Rainy: (137, 164, 196, 971)	Dry: (127, 204, 379, 539) Rainy: (52, 73, 78, 103)	(187, 221, 380, 667) (114, 154, 268, 714)	(7, 19, 51, 52) (18, 21, 62, 91)	Dry: (245, 273, 475, 404) Rainy: (722, 605, 864, 768)
$M^r_{s,a}$ ‡	SETA	Dry: (441, 515, 542, 926) Rainy: (300, 349, 441, 885)	Dry: (466, 423, 575, 670) Rainy: (675, 679, 926, 811)	Dry: (126, 164, 190, 916) Rainy: (137, 164, 196, 970)	Dry: (125, 204, 379, 538) Rainy: (51, 73, 78, 102)	(185, 221, 378, 664) (112, 149, 258, 709)	(7, 19, 51, 50) (18, 20, 61, 90)	Dry: (237, 269, 454, 389) Rainy: (705, 594, 839, 750)
$F^r_{s,a}$ ‡	SETA	Dry: (0, 0, 2, 3) Rainy: (0, 2, 2, 2)	Dry: (1, 4, 12, 4) Rainy: (3, 2, 14, 11)	Dry: (0, 0, 3, 1) Rainy: (0, 0, 2, 1)	Dry: (2, 6, 27, 7) Rainy: (0, 2, 15, 1)	(0, 0, 10, 24) (0, 1, 6, 8)	(0, 0, 0, 0) (0, 0, 1, 0)	Dry: (0, 6, 20, 3) Rainy: (2, 4, 27, 3)
$G_{s,a}$ ‡	HCUS	(68,182,789,2352)	(26,65,199,466)	(90,160,433,392)	(117,162,622,1616)	(119,270,812,2100)	(81,137,248,200)	(4,16,113,464)
$H_{s,a}$ ‡	HCUS	(105,292,1201,3454)	(56,154,474,1181)	(54,103,261,238)	(353,672,2218,5495)	(193,452,1504,4766)	(238,356,664,651)	(77,175,983,3393)
$J^r_{c,a}$ ‡	HCUS	Dry: (49,96,387,1585) Rainy: (40,108,461,1113)	Dry: (96,229,1023,1999) Rainy: (96,229,1023,1999)	Dry: (77,132,360,418), Rainy: (77,132,360,418)	Dry: (67,118,408,868) Rainy: (66,106,356,985)	(90,181,477,1698) (114,249,640,666)	(90,181,477,1698) (114,249,640,666)	Dry: (7,23,112,457) Rainy: (4,12,73,303)
$K^r_{c,a}$ ‡	HCUS	Dry: (98,192,774,3170), Rainy: (63,193,806,1934)	Dry: (207,538,2258,4910) Rainy: (207,538,2258,4910)	Dry: (200,289,777,782) Rainy: (200,289,777,782)	Dry: (453,1157,3250,3626) Rainy: (117,162,622,1616)	(359,706,1720,5719) (274,651,1552,2255)	(359,706,1720,5719) (274,651,1552,2255)	Dry: (58,121,724,2523) Rainy: (44,115,574,2041)
N^s_{elig}	SETA's admission log-book review	7460	13064	5354	820	4240	334	3770
N^s_{enroll}	SETA's admission log-book review	1229	5080	2235	452	2647	504	1271

HCUS, healthcare utilization survey; UNDP, United Nations Development Program

† For the healthcare utilization surveys conducted in the Democratic Republic of the Congo and Ethiopia, it was not possible to differentiate usage levels by season, so age-group specific usage levels were assumed to not vary with season for sites in these two countries.

‡ Values are presented for each of four age groups in the following order: < 2 years, 2 to 4 years, 5 to 14 years, and 15 years and older.

*indicates an assumed value

C.1 Code for the MultiBUGS Model for *S.* Typhi incidence

```
model{

## Note that '_r'/'_d' in a variable/parameter name delineates rainy versus dry season specific
##entities

##Data definition
##Loop through the four (4) age groups

##Epsilon is defined as a small adjustment factor to avoid undefined derived quantities (for
##example, due to attempted division by 0)

epsilon<-0.0000001

for(b in 1:4) {

	##DEFINE PATHOGEN-customize the next two lines to for target pathogen or pathogen group
	bc_pos_y_r[b] <- bc_pos_y_typhi_r[b]
	bc_pos_y_d[b] <- bc_pos_y_typhi_d[b]

	bc_pos_r_y[b]<-bc_pos_y_r[b]+(1-step(-1*epsilon+bc_pos_y_r[b]))*epsilon
	bc_pos_d_y[b]<-bc_pos_y_d[b]+(1-step(-1*epsilon+bc_pos_y_d[b]))*epsilon

	bc_pos_r_n[b]<-bc_pos_n_r[b]+(1-step(-1*epsilon+bc_pos_n_r[b]))*epsilon+(1-step(-
1*epsilon+bc_pos_n_r[b]-bc_pos_y_r[b]))*epsilon

	bc_pos_d_n[b]<-bc_pos_n_d[b]+(1-step(-1*epsilon+bc_pos_n_d[b]))*epsilon+(1-step(-
1*epsilon+bc_pos_n_d[b]-bc_pos_y_d[b]))*epsilon

	bc_perform_r_y[b]<-bc_perform_y_r[b]+(1-step(-1*epsilon+bc_perform_y_r[b]))*epsilon
	bc_perform_d_y[b]<-bc_perform_y_d[b]+(1-step(-1*epsilon+bc_perform_y_d[b]))*epsilon

	bc_perform_r_n[b]<-bc_perform_N_r[b]+(1-step(-1*epsilon+bc_perform_N_r[b]))*epsilon+(1-step(-
1*epsilon+bc_perform_N_r[b]-bc_perform_y_r[b]))*epsilon
	bc_perform_d_n[b]<-bc_perform_N_d[b]+(1-step(-1*epsilon+bc_perform_N_d[b]))*epsilon+(1-step(-
1*epsilon+bc_perform_N_d[b]-bc_perform_y_d[b]))*epsilon



}

## PRIOR DISTRIBUTIONS

##  1/variance of h and p
tau_h ~ dgamma(1,1)
tau_p ~ dgamma(1,1)

## Sensitivity of Blood Culture for S. Typhi
sens_bc ~ dnorm(0.6,400)

## Annual population estimates
tau_pop ~ dgamma(1,1)
g_rt[1] ~ dnorm(wb_growth[1],tau_pop)
pop[1] <- ref_pop*(1+sign_grt[1]*g_rt[1]/100)
for(y in 2:Y) {
```

```
        g_rt[y] ~ dnorm(wb_growth[y],tau_pop)
        pop[y] <- (1-abs(sign_grt[y]))*ref_pop+abs(sign_grt[y])*pop[y-1]*(1+sign_grt[y]*g_rt[y]/100)
}

        ##Probability of being enrolled into SETA upon seeking care at a participating HCF

        logit_enroll <- log((enroll_y/enroll_n)/(1-(enroll_y/enroll_n)))
        tau_logit_enroll <- 3/pow(enroll_y/enroll_n,2)
        lnpr_enroll ~ dlogis(logit_enroll,tau_logit_enroll)
        pr_enroll <-  ilogit(lnpr_enroll)


for(a in 1:A) {

        ##Age group specific population estimates

        for(y in 1:Y) {
                pop_a[y,a]<-pop[y]*pop_a_prop[a]*include[y]
        }
        py[a]<-sum(pop_a[1:Y,a])

        for(b in Ast[a]:Aed[a]) {
                py_a_cat[b] <-
sum(pop_a[1:Y,a])*(bc_perform_r_n[b]+bc_perform_d_n[b])/(sum(bc_perform_r_n[Ast[a]:Aed[a]])+sum(bc_perfo
rm_d_n[Ast[a]:Aed[a]]))
        }

        ##Generic seasonal probability of seeking care at a SETA facility versus another HCF when febrile

        lgor_seta_hcu_r[a] <- log((seta_hcf_r_y[a])*((seta_hcf_d_n[a]+seta_hcf_r_n[a])-
(seta_hcf_d_y[a]+seta_hcf_r_y[a]))/((seta_hcf_d_y[a]+seta_hcf_r_y[a])*(seta_hcf_r_n[a]-(seta_hcf_r_y[a]))))

        var_lgor_seta_hcu_r[a] <- 1/(seta_hcf_r_y[a])+1/((seta_hcf_d_n[a]+seta_hcf_r_n[a])-
(seta_hcf_d_y[a]+seta_hcf_r_y[a]))+1/(seta_hcf_d_y[a]+seta_hcf_r_y[a])+1/(seta_hcf_r_n[a]-(seta_hcf_r_y[a]))

        lgor_seta_hcu_d[a] <- log((seta_hcf_d_y[a])*((seta_hcf_r_n[a]+seta_hcf_d_n[a])-
(seta_hcf_r_y[a]+seta_hcf_d_y[a]))/((seta_hcf_r_y[a]+seta_hcf_d_y[a])*(seta_hcf_d_n[a]-(seta_hcf_d_y[a]))))

        var_lgor_seta_hcu_d[a] <- 1/(seta_hcf_d_y[a])+1/((seta_hcf_d_n[a]+seta_hcf_r_n[a])-
(seta_hcf_d_y[a]+seta_hcf_r_y[a]))+1/(seta_hcf_d_y[a]+seta_hcf_r_y[a])+1/(seta_hcf_d_n[a]-(seta_hcf_d_y[a]))

        logit_seta_hcu_r[a] <- logit(seta_hcf_y[a]/seta_hcf_n[a])+lgor_seta_hcu_r[a]

        tau_logit_seta_hcu_r[a] <- 1/(pow(seta_hcf_y[a] / seta_hcf_n[a],2) / 3 + var_lgor_seta_hcu_r[a])

        lnpr_seta_hcu_r[a] ~ dlogis(logit_seta_hcu_r[a],tau_logit_seta_hcu_r[a])

        pr_seta_hcu_r[a] <-  ilogit(lnpr_seta_hcu_r[a])

        logit_seta_hcu_d[a] <- logit(seta_hcf_y[a]/seta_hcf_n[a])+lgor_seta_hcu_d[a]

        tau_logit_seta_hcu_d[a] <- 1/(pow(seta_hcf_y[a] / seta_hcf_n[a],2) / 3 +var_lgor_seta_hcu_d[a])

        lnpr_seta_hcu_d[a] ~ dlogis(logit_seta_hcu_d[a],tau_logit_seta_hcu_d[a])

        pr_seta_hcu_d[a] <-  ilogit(lnpr_seta_hcu_d[a])
```

```
##Seasonal probability that a blood culture will be performed once enrolled in SETA
for(b in Ast[a]:Aed[a]) {
    logit_bc_perform_r[b] <- logit(bc_perform_r_y[b]/bc_perform_r_n[b])
    tau_logit_bc_perform_r[b] <- 3 / pow(bc_perform_r_y[b] / bc_perform_r_n[b], 2)
    lnpr_bc_perform_r[b] ~ dlogis(logit_bc_perform_r[b],tau_logit_bc_perform_r[b])
    pr_bc_perform_r[b] <-  ilogit(lnpr_bc_perform_r[b])
    logit_bc_perform_d[b] <- logit(bc_perform_d_y[b] / bc_perform_d_n[b])
    tau_logit_bc_perform_d[b] <- 3 / pow(bc_perform_d_y[b] / bc_perform_d_n[b],2)
    lnpr_bc_perform_d[b] ~ dlogis(logit_bc_perform_d[b],tau_logit_bc_perform_d[b])
    pr_bc_perform_d[b] <-  ilogit(lnpr_bc_perform_d[b])
    ##Proportion of blood cultures positive for S. Typhi
    logit_bc_pos_r[b] <- logit(bc_pos_r_y[b] / bc_pos_r_n[b])
    tau_logit_bc_pos_r[b] <- 3 / pow(bc_pos_r_y[b] / bc_pos_r_n[b],2)
    lnpr_bc_pos_r[b] ~ dlogis(logit_bc_pos_r[b],tau_logit_bc_pos_r[b])
    pr_bc_pos_r[b] <-  ilogit(lnpr_bc_pos_r[b])
    logit_bc_pos_d[b] <- logit(bc_pos_d_y[b]/bc_pos_d_n[b])
    tau_logit_bc_pos_d[b] <- 3 / pow(bc_pos_d_y[b] / bc_pos_d_n[b],2)
    lnpr_bc_pos_d[b] ~ dlogis(logit_bc_pos_d[b],tau_logit_bc_pos_d[b])
    pr_bc_pos_d[b] <-  ilogit(lnpr_bc_pos_d[b])
    y_seta_bc_r[b] <- bc_pos_r_y[b]/sens_bc
    y_seta_bc_d[b] <- bc_pos_d_y[b]/sens_bc
    y_seta_bc[b] <-y_seta_bc_d[b] +y_seta_bc_r[b]
    y_seta_no_bc_r[b] <- (bc_perform_r_n[b] - bc_perform_r_y[b])*pr_bc_pos_r[b]/sens_bc
    y_seta_no_bc_d[b] <- (bc_perform_d_n[b] - bc_perform_d_y[b])*pr_bc_pos_d[b]/sens_bc
    y_seta_no_bc[b] <- y_seta_no_bc_d[b] + y_seta_no_bc_r[b]
    y_elig_no_enroll_r[b] <- (bc_perform_r_n[b]/pr_enroll - bc_perform_r_n[b]) * pr_bc_pos_r[b] / sens_bc
    y_elig_no_enroll_d[b] <-  (bc_perform_d_n[b] / pr_enroll - bc_perform_d_n[b]) * pr_bc_pos_d[b] /
sens_bc
```

```
		y_elig_no_enroll[b] <-y_elig_no_enroll_d[b]+y_elig_no_enroll_r[b]

		y_non_seta_hcf_r[b] <- (bc_perform_r_n[b]/pr_enroll/pr_seta_hcu_r[a]*(1-
pr_seta_hcu_r[a]))*pr_bc_pos_r[b]/sens_bc

		y_non_seta_hcf_d[b] <- (bc_perform_d_n[b]/pr_enroll/pr_seta_hcu_d[a]*(1-
pr_seta_hcu_d[a]))*pr_bc_pos_d[b]/sens_bc

		y_non_seta_hcf[b] <- y_non_seta_hcf_d[b] + y_non_seta_hcf_r[b]

		cases[b]<- (1-
equals(bc_pos_y_d[b]+bc_pos_y_r[b],0))*(y_seta_bc[b]+y_seta_no_bc[b]+y_elig_no_enroll[b]+y_non_seta_hcf[b]
)

		ir[b]<-(1-equals(bc_pos_y_d[b]+bc_pos_y_r[b],0))*cases[b]/py_a_cat[b]*100000

		py_t[b]<-(1-equals(bc_pos_y_d[b]+bc_pos_y_r[b],0))*py_a_cat[b]
		cases_t[b]<-(1-equals(bc_pos_y_d[b]+bc_pos_y_r[b],0))*cases[b]

		elig_N[b]<-(bc_perform_d_n[b]+bc_perform_r_n[b])/pr_enroll

		prop_seta_bc[b]<-y_seta_bc[b]/(cases[b]+epsilon)
		prop_seta_no_bc[b]<-y_seta_no_bc[b]/(cases[b]+epsilon)
		prop_non_seta_hcf[b]<-y_non_seta_hcf[b] / (cases[b]+epsilon)
}
}
cases_total<-sum(cases_t[1:4])
py_total<-sum(py_a_cat[1:4])
ir_total<-cases_total/py_total*100000

elig_total<-sum(elig_N[1:4])
refus_total <- sum(elig_N[1:4]) - (sum(bc_perform_d_n[1:4]) + sum(bc_perform_r_n[1:4]) )
}
```

C.2. Code for each dataset

```
#DATA
#Burkina Faso - Nioko+Polesgo
list(
Y=5,
A=4,
ref_pop=27148,
enroll_y=1229,
enroll_n=7460,
sign_grt=c(-1,0,1,1,1),
wb_growth=c(2.9,0,2.9,2.9,2.9),
include=c(0.66,1,1,1,1),
pop_a_prop=c(0.12278938,0.10537190,0.23856958,0.53326914),
seta_hcf_d_n=c(98,192,774,3170),
seta_hcf_d_y=c(49,96,387,1585),
seta_hcf_r_n=c(63,193,806,1934),
seta_hcf_r_y=c(40,108,461,1113),
seta_hcf_y=c(68,182,789,2352),
seta_hcf_n=c(105,292,1201,3454),
bc_perform_N_d =
c(454, 516, 548, 947),
bc_perform_y_d =
c(441, 515, 542, 926),
bc_pos_n_d =
c(441, 515, 542, 926),
bc_pos_y_typhi_d =
c(0, 0, 2, 3),
bc_perform_N_r =
c(302, 352, 449, 904),
bc_perform_y_r =
c(300, 349, 441, 885),
bc_pos_n_r =
c(300, 349, 441, 885),
bc_pos_y_typhi_r =
c(0, 2, 2, 2),
Ast=c(1,2,3,4),
Aed=c(1,2,3,4)
)

#DATA
#DRC - Kavuaya and Nkandu 1
list(
list(
Y=3,
A=4,
ref_pop=54731,
enroll_y=5080,
enroll_n=13064,
sign_grt=c(0,1,1),
wb_growth=c(3.3,3.2,3.2),
include=c(1,1,1),
pop_a_prop=c(0.06922936,0.09671545,0.29178921,0.54226598),
seta_hcf_d_n=c(207,538,2258,4910),
seta_hcf_d_y=c(96,229,1023,1999),
seta_hcf_r_n=c(207,538,2258,4910),
seta_hcf_r_y=c(96,229,1023,1999),
```

```
seta_hcf_y=c(26,65,199,466),
seta_hcf_n=c(56,154,474,1181),
bc_perform_N_d =
c(469, 425, 578, 671),
bc_perform_y_d =
c(466, 423, 575, 670),
bc_pos_n_d =
c(466, 423, 575, 670),
bc_pos_y_typhi_d =
c(1, 4, 12, 4),
bc_perform_N_r =
c(681, 682, 930, 811),
bc_perform_y_r =
c(675, 679, 927, 811),
bc_pos_n_r =
c(675, 679, 927, 811),
bc_pos_y_typhi_r =
c(3, 2, 14, 11),
Ast=c(1,2,3,4),
Aed=c(1,2,3,4)
)

#DATA
#Ethiopia - Sodo
list(
Y=3,
A=4,
ref_pop=120288,
enroll_y=2235,
enroll_n=5354,
sign_grt=c(0,1,1),
wb_growth=c(2.7,2.6,2.6),
include=c(0.33,1,1),
pop_a_prop=c(0.05121874,0.10488993,0.32261738,0.52127394),
seta_hcf_d_n=c(200,289,777,782),
seta_hcf_d_y=c(77,132,360,418),
seta_hcf_r_n=c(200,289,777,782),
seta_hcf_r_y=c(77,132,360,418),
seta_hcf_n=c(90,160,433,392),
seta_hcf_y=c(54,103,261,238),
bc_perform_N_d =
c(126, 164, 190, 916),
bc_perform_y_d =
c(126, 164, 190, 916),
bc_pos_n_d =
c(126, 164, 190, 916),
bc_pos_y_typhi_d =
c(0, 0, 3, 1),
bc_perform_N_r =
c(137, 164, 196, 971),
bc_perform_y_r =
c(137, 164, 196, 970),
bc_pos_n_r =
c(137, 164, 196, 970),
bc_pos_y_typhi_r =
c(0, 0, 2, 1),
```

```
Ast=c(1,2,3,4),
Aed=c(1,2,3,4)
)


#DATA
#Ghana - Agogo
list(
Y=10,
A=4,
ref_pop=140694,
enroll_y=452,
enroll_n=820,
sign_grt=c(0,1,1,1,1,1,1,1,1,1),
wb_growth=c(1,2.5,2.4,2.4,2.3,2.3,2.2,2.2,2.1,2.1),
include=c(0,0,0,0,0,0,0.66,1,1,1),
pop_a_prop=c(0.08249108,0.059441056,0.253159339,0.604908525),
seta_hcf_d_n=c(453,1157,3250,3626),
seta_hcf_d_y=c(67,118,408,868),
seta_hcf_r_n=c(305,671,1908,5173),
seta_hcf_r_y=c(66,106,356,985),
seta_hcf_n=c(353,672,2218,5495),
seta_hcf_y=c(117,162,622,1616),
bc_perform_N_d =
c(127, 204, 379, 539),
bc_perform_y_d =
c(125, 204, 379, 538),
bc_pos_n_d =
c(125, 204, 379, 538),
bc_pos_y_typhi_d =
c(2, 6, 27, 7),
bc_perform_N_r =
c(52, 73, 78, 103),
bc_perform_y_r =
c(51, 73, 78, 102),
bc_pos_n_r =
c(51, 73, 78, 102),
bc_pos_y_typhi_r =
c(0, 2, 15, 1),
Ast=c(1,2,3,4),
Aed=c(1,2,3,4)
)


#DATA
#Madagascar – Imerintsiatosika
list(
Y=4,
A=4,
ref_pop=44669,
enroll_y=2647,
enroll_n=4240,
sign_grt=c(0,1,1,1),
wb_growth=c(1,1.6,1.6,1.6),
include=c(0.91,1,1,1),
pop_a_prop=c(0.08018984,0.09959927,0.17036423,0.64984665),
```

```
seta_hcf_d_n=c(359,706,1720,5719),
seta_hcf_d_y=c(90,181,477,1698),
seta_hcf_r_n=c(274,651,1552,2255),
seta_hcf_r_y=c(114,249,640,666),
seta_hcf_n=c(193,452,1504,4766),
seta_hcf_y=c(119,270,812,2100),
bc_perform_N_d =
c(187, 221, 380, 667),
bc_perform_y_d =
c(185, 221, 378, 664),
bc_pos_n_d =
c(185, 221, 378, 664),
bc_pos_y_typhi_d =
c(0, 0, 10, 24),
bc_perform_N_r =
c(114, 154, 268, 714),
bc_perform_y_r =
c(112, 149, 258, 709),
bc_pos_n_r =
c(112, 149, 258, 709),
bc_pos_y_typhi_r =
c(0, 1, 6, 8),
Ast=c(1,2,3,4),
Aed=c(1,2,3,4)
)

#DATA
#Madagascar – Mahajanga
list(
Y=4,
A=4,
ref_pop=5475,
enroll_y=334,
enroll_n=504,
sign_grt=c(0,1,1,1),
wb_growth=c(1,1.6,1.6,1.6),
include=c(0.58,1,1,1),
pop_a_prop=c(0.06730330,0.08565874,0.26401183,0.58302613),
seta_hcf_d_n=c(359,706,1720,5719),
seta_hcf_d_y=c(90,181,477,1698),
seta_hcf_r_n=c(274,651,1552,2255),
seta_hcf_r_y=c(114,249,640,666),
seta_hcf_n=c(238,356,664,651),
seta_hcf_y=c(81,137,248,200),
bc_perform_N_d =
c(7, 19, 51, 52),
bc_perform_y_d =
c(7, 19, 51, 50),
bc_pos_n_d =
c(7, 19, 51, 50),
bc_pos_y_typhi_d =
c(0, 0, 0, 0),
bc_perform_N_r =
c(18, 21, 62, 91),
bc_perform_y_r =
c(18, 20, 61, 90),
```

```
bc_pos_n_r =
c(18, 20, 61, 90),
bc_pos_y_typhi_r =
c(0, 0, 1, 0),
Ast=c(1,2,3,4),
Aed=c(1,2,3,4)
)

#DATA
#Nigeria - Ibadan
list(
Y=15,
A=4,
ref_pop=1343147,
enroll_y=1271,
enroll_n=3770,
sign_grt=c(0,1,1,1,1,1,1,1,1,1,1,1,1,1,1),
wb_growth=c(1.0,4.8,4.8,4.8,4.7,4.7,4.6,4.6,4.5,4.4,4.4,4.3,4.23,4.23,4.23),
include=c(0,0,0,0,0,0,0,0,0,0,0,0.91,1,1,1),
pop_a_prop=c(0.04190513,0.08921231,0.22756705,0.64131551),
seta_hcf_d_n=c(58,121,724,2523),
seta_hcf_d_y=c(7,23,112,457),
seta_hcf_r_n=c(44,115,574,2041),
seta_hcf_r_y=c(4,12,73,303),
seta_hcf_n=c(77,175,983,3393),
seta_hcf_y=c(4,16,113,464),
bc_perform_N_d =
c(245, 273, 475, 404),
bc_perform_y_d =
c(237, 269, 455, 389),
bc_pos_n_d =
c(237, 269, 455, 389),
bc_pos_y_typhi_d =
c(0, 6, 20, 3),
bc_perform_N_r =
c(722, 605, 864, 768),
bc_perform_y_r =
c(705, 594, 840, 750),
bc_pos_n_r =
c(705, 594, 840, 750),
bc_pos_y_typhi_r =
c(2, 4, 27, 3),
Ast=c(1,2,3,4),
Aed=c(1,2,3,4)
)
```

C3. Batch script for running analysis in MultiBUGS

```
modelDisplay('log')
##Enter the directory address for the data folder.  All files are expected to be saved in the same folder.
##The directory address needs to be bracketed by single quotes, and for Windows machines the '/' symbol can be
##used to separate directory and sub-directory names.
##The folder should contain a file containing the model with file name: 'model_S_Typhi.odc' and
## one data file per site (use the file names listed below in each modelData() statement.

modelSetWD(##DIRECTORY ADDRESS##)

##Burkina Faso Nioko and Polesgo##
modelCheck('model_S_Typhi.odc')
modelData('bk_nioko_polesgo_data.odc')
modelCompile(3)
modelSetRN(1)
modelGenInits()
samplesSet('ir')
samplesSet('py_a_cat')
samplesSet('cases')
samplesSet('cases_total')
samplesSet('ir_total')
samplesSet('py_total')
samplesSet('pr_seta_hcu_d')
samplesSet('pr_seta_hcu_r')
samplesSet('sens_bc')
samplesSet('pr_enroll')
samplesSet('pr_bc_perform_r')
samplesSet('pr_bc_perform_d')
samplesSet('elig_total')
samplesSet('refus_total')
modelUpdate(30000)
samplesBeg(5001)
samplesEnd(30000)
samplesFirstChain(1)
samplesLastChain(3)
samplesThin(50)
samplesStats('*')

##DRC Kavuaya/Nkandu-1##
modelCheck('model_S_Typhi.odc')
modelData('drc_kavuaya_nkandu.odc')
modelCompile(3)
modelSetRN(1)
modelGenInits()
samplesSet('ir')
samplesSet('py_a_cat')
samplesSet('cases')
samplesSet('cases_total')
samplesSet('ir_total')
samplesSet('py_total')
samplesSet('pr_seta_hcu_d')
samplesSet('pr_seta_hcu_r')
samplesSet('sens_bc')
samplesSet('pr_enroll')
samplesSet('pr_bc_perform_r')
samplesSet('pr_bc_perform_d')
```

```
samplesSet('elig_total')
samplesSet('refus_total')
modelUpdate(30000)
samplesBeg(5001)
samplesEnd(30000)
samplesFirstChain(1)
samplesLastChain(3)
samplesThin(50)
samplesStats('*')


##Ethiopia Sodo##
modelCheck('model_S_Typhi.odc')
modelData('et_sodo_data.odc')
modelCompile(3)
modelSetRN(1)
modelGenInits()
samplesSet('ir')
samplesSet('py_a_cat')
samplesSet('cases')
samplesSet('cases_total')
samplesSet('ir_total')
samplesSet('py_total')
samplesSet('pr_seta_hcu_d')
samplesSet('pr_seta_hcu_r')
samplesSet('sens_bc')
samplesSet('pr_enroll')
samplesSet('pr_bc_perform_r')
samplesSet('pr_bc_perform_d')
samplesSet('elig_total')
samplesSet('refus_total')
modelUpdate(30000)
samplesBeg(5001)
samplesEnd(30000)
samplesFirstChain(1)
samplesLastChain(3)
samplesThin(50)
samplesStats('*')


##Ghana Agogo##
modelCheck('model_S_Typhi.odc')
modelData('gh_agogo_data.odc')
modelCompile(3)
modelSetRN(1)
modelGenInits()
samplesSet('ir')
samplesSet('py_a_cat')
samplesSet('cases')
samplesSet('cases_total')
samplesSet('ir_total')
samplesSet('py_total')
samplesSet('pr_seta_hcu_d')
samplesSet('pr_seta_hcu_r')
samplesSet('sens_bc')
samplesSet('pr_enroll')
```

```
samplesSet('pr_bc_perform_r')
samplesSet('pr_bc_perform_d')
samplesSet('elig_total')
samplesSet('refus_total')
modelUpdate(30000)
samplesBeg(5001)
samplesEnd(30000)
samplesFirstChain(1)
samplesLastChain(3)
samplesThin(50)
samplesStats('*')

##Madagascar Imerintsiatosika##
modelCheck('model_iNTS_corrected.odc')
modelData('md_imer_data.odc')
modelCompile(3)
modelSetRN(1)
modelGenInits()
samplesSet('ir')
samplesSet('py_a_cat')
samplesSet('cases')
samplesSet('cases_total')
samplesSet('ir_total')
samplesSet('py_total')
samplesSet('pr_seta_hcu_d')
samplesSet('pr_seta_hcu_r')
samplesSet('sens_bc')
samplesSet('pr_enroll')
samplesSet('pr_bc_perform_r')
samplesSet('pr_bc_perform_d')
samplesSet('elig_total')
samplesSet('refus_total')
modelUpdate(30000)
samplesBeg(5001)
samplesEnd(30000)
samplesFirstChain(1)
samplesLastChain(3)
samplesThin(50)
samplesStats('*')

##Madagascar Mahajanga##
modelCheck('model_iNTS_corrected.odc')
modelData('md_maha_data.odc')
modelCompile(3)
modelSetRN(1)
modelGenInits()
samplesSet('ir')
samplesSet('py_a_cat')
samplesSet('cases')
samplesSet('cases_total')
samplesSet('ir_total')
samplesSet('py_total')
samplesSet('pr_seta_hcu_d')
samplesSet('pr_seta_hcu_r')
samplesSet('sens_bc')
samplesSet('pr_enroll')
```

```
samplesSet('pr_bc_perform_r')
samplesSet('pr_bc_perform_d')
samplesSet('elig_total')
samplesSet('refus_total')
modelUpdate(30000)
samplesBeg(5001)
samplesEnd(30000)
samplesFirstChain(1)
samplesLastChain(3)
samplesThin(50)
samplesStats('*')

##Nigeria Ibadan##
modelCheck('model_S_Typhi.odc')
modelData('ni_ibadan_data.odc')
modelCompile(3)
modelSetRN(1)
modelGenInits()
samplesSet('ir')
samplesSet('py_a_cat')
samplesSet('cases')
samplesSet('cases_total')
samplesSet('ir_total')
samplesSet('py_total')
samplesSet('pr_seta_hcu_d')
samplesSet('pr_seta_hcu_r')
samplesSet('sens_bc')
samplesSet('pr_enroll')
samplesSet('pr_bc_perform_r')
samplesSet('pr_bc_perform_d')
samplesSet('elig_total')
samplesSet('refus_total')
modelUpdate(30000)
samplesBeg(5001)
samplesEnd(30000)
samplesFirstChain(1)
samplesLastChain(3)
samplesThin(50)
samplesStats('*')
modelSaveLog('results_S_Typhi.txt')
modelQuit('yes')
```

C4. R code for post processing the MultiBUGS output

```
rm(list=ls())
##The next line sets the address of the directory where the MultiBUGS input files and output file
##(results_S_Typhi.txt) are saved.
setwd('##DIRECTORY ADDRESS##')
input<-readLines(con="results_S_Typhi.txt")

input<-input[grepl("\t",input)]

g<-0
z1<-c()
z2<-c()
for(j in 1:length(input)) {
		z0<-unlist(strsplit(input[j],"\t"))
		z0<-z0[(z0=="")==F]
		if(grepl("mean",input[j])==F) {
				z1<-rbind(z1,z0,deparse.level=0)
		}
		g<-ifelse(grepl("mean",input[j]),g+1,g)
		if(grepl("mean",input[j])==F) {
				z2<-c(z2,g)
		}
}
z1<-cbind(z1,z2,agegrp=rep(0,length(z2)))
colnames(z1)<-c("param",unlist(strsplit(input[1],"\t"))[3:length(unlist(strsplit(input[1],"\t")))],"area_id","agegrp")


z4<-cbind(country=c("Burkina Faso","Democratic Republic of the
Congo","Ethiopia","Ghana","Madagascar","Madagascar","Nigeria"),
	site=c("Nioko and Polesgo","Kavuaya and Nkandu
1","Sodo","Agogo","Imerintsiatosika","Mahajanga","Ibadan"),
	area_id=seq(1,7))

z1<-merge(z1,z4)
z1$agegrp<-as.vector(z1$agegrp)
z1$agegrp[grepl('\\[1\\]',z1$param)]<-1
z1$agegrp[grepl('\\[2\\]',z1$param)]<-2
z1$agegrp[grepl('\\[3\\]',z1$param)]<-3
z1$agegrp[grepl('\\[4\\]',z1$param)]<-4

z1$agegrp[grepl('total',z1$param)]<-5


for(i in 1:4) {
		z1$param<-sub(paste('\\[',i,'\\]',sub=''),"",z1$param)
}

z5<-z1[,c("area_id","country","site","agegrp","mean","sd","val2.5pc","val97.5pc","param")]

casemat<-
cbind(z1[grepl("case",z1$param),c("area_id","country","site","agegrp")],casetxt=paste(format(round(as.numeric(as.
vector(z1$mean[grepl("case",z1$param)])),0),nsmall=0,big.mark=",",trim=T),"
(",format(round(as.numeric(as.vector(z1$val2.5pc[grepl("case",z1$param)])),0),nsmall=0,big.mark=",",trim=T),";
",format(round(as.numeric(as.vector(z1$val97.5pc[grepl("case",z1$param)])),0),nsmall=0,big.mark=",",trim=T),")",
sep=""))
```

```
pymat<-
cbind(z1[grepl("py",z1$param),c("area_id","country","site","agegrp")],pytext=paste(format(round(as.numeric(as.vector(z1$mean[grepl("py",z1$param)])),0),nsmall=0,big.mark=",",trim=T),"
(",format(round(as.numeric(as.vector(z1$val2.5pc[grepl("py",z1$param)])),0),nsmall=0,big.mark=",",trim=T),";
",format(round(as.numeric(as.vector(z1$val97.5pc[grepl("py",z1$param)])),0),nsmall=0,big.mark=",",trim=T),")",sep=""))
irmat<-
cbind(z1[grepl("ir",z1$param),c("area_id","country","site","agegrp")],irtext=paste(format(round(as.numeric(as.vector(z1$mean[grepl("ir",z1$param)])),0),nsmall=0,big.mark=",",trim=T),"
(",format(round(as.numeric(as.vector(z1$val2.5pc[grepl("ir",z1$param)])),0),nsmall=0,big.mark=",",trim=T),";
",format(round(as.numeric(as.vector(z1$val97.5pc[grepl("ir",z1$param)])),0),nsmall=0,big.mark=",",trim=T),")",sep=""))

finmat<-merge(casemat,pymat,all.x=T,all.y=T)
finmat<-merge(finmat,irmat,all.x=T,all.y=T)
finmat<-as.matrix(finmat)

finmat[is.na(finmat)]<-'-'
finmat[finmat=="0 (0, 0)"]<-'-'



write.csv(finmat[finmat[,4]!="0",],"table_S_Typhi2.csv")
```
